# Developing a Hybrid Risk Assessment Tool for Familial Hypercholesterolemia: A Machine Learning Study of Chinese Arteriosclerotic Cardiovascular Disease Patients

**DOI:** 10.3389/fcvm.2022.893986

**Published:** 2022-08-03

**Authors:** Lei Wang, Jian Guo, Zhuang Tian, Samuel Seery, Ye Jin, Shuyang Zhang

**Affiliations:** ^1^State Key Laboratory of Complex Severe and Rare Diseases, Department of Cardiology, Peking Union Medical College Hospital, Chinese Academy of Medical Sciences and Peking Union Medical College, Beijing, China; ^2^State Key Laboratory of Complex Severe and Rare Diseases, Medical Research Center, Peking Union Medical College Hospital, Chinese Academy of Medical Sciences and Peking Union Medical College, Beijing, China; ^3^Department of Humanities and Social Sciences, Peking Union Medical College Hospital, Chinese Academy of Medical Sciences and Peking Union Medical College, Beijing, China

**Keywords:** familial hypercholesterolemia (FH), risk assessment, DLCN, early detection and prevention, hybrid diagnosis

## Abstract

**Background:**

Familial hypercholesterolemia (FH) is an autosomal-dominant genetic disorder with a high risk of premature arteriosclerotic cardiovascular disease (ASCVD). There are many alternative risk assessment tools, for example, DLCN, although their sensitivity and specificity vary among specific populations. We aimed to assess the risk discovery performance of a hybrid model consisting of existing FH risk assessment tools and machine learning (ML) methods, based on the Chinese patients with ASCVD.

**Materials and Methods:**

In total, 5,597 primary patients with ASCVD were assessed for FH risk using 11 tools. The three best performing tools were hybridized through a voting strategy. ML models were set according to hybrid results to create a hybrid FH risk assessment tool (HFHRAT). PDP and ICE were adopted to interpret black box features.

**Results:**

After hybridizing the mDLCN, Taiwan criteria, and DLCN, the HFHRAT was taken as a stacking ensemble method (AUC_class[94.85 ± 0.47], AUC_prob[98.66 ± 0.27]). The interpretation of HFHRAT suggests that patients aged <75 years with LDL-c >4 mmol/L were more likely to be at risk of developing FH.

**Conclusion:**

The HFHRAT has provided a median of the three tools, which could reduce the false-negative rate associated with existing tools and prevent the development of atherosclerosis. The hybrid tool could satisfy the need for a risk assessment tool for specific populations.

## Introduction

Familial hypercholesterolemia (FH) (1) is an autosomal-dominant genetic disorder with a high risk of premature arteriosclerotic cardiovascular disease (ASCVD). Underdiagnosis and indeed undertreatment create problems for patients with FH around the world (2). Unfortunately, the global rate of FH diagnosis is only approximately 1% (3), despite having an estimated prevalence of 1:313 (4) in the general population. There is, however, increasing interest in FH due to growing concerns about the rising levels of cholesterol in diets (5). We know that early lipid-lowering therapies hinder the development of ASCVD (6); however, most cases are identified only after encountering an ASCVD event. Underdiagnosis and undertreatment of FH are partially due to the fact that we do not have an effective gold standard to identify high-risk patients at an early stage.

At present, genetic testing is the gold standard for FH (7). However, the high costs of genetic testing and counseling have not yet been covered by social medical insurance, which has limited their application for FH diagnosis in clinical practice (8). To address these issues, researchers have developed phenotypic tools which intercalate an assessment of clinical features, family history, and genetic test results. The Dutch Lipid Clinic Network (DLCN) (9), Simon Broome Register (SBR) (10), and “Make Early Diagnosis to Prevent Early Deaths” (MEDPED) are three commonly used tools in clinical practice (11). These three tools are advocated for clinical use in many different countries (12–14), although subtle differences within (and between) populations have been acknowledged.

The FH diagnosis rate is <0.1% in China (3). The phenotypic tools were applied; however, DLCN had low specificity and SBR had low sensitivity (15) in mainland Chinese populations. MEDPED is also complicated because of difficulties in collecting family histories (16). Recently, researchers have devised novel tools for Chinese patients mainly by modifying the DLCN model. Even though these are based on comparatively lower levels of LDL cholesterol in mainland Chinese populations or to overcome difficulties in collecting family histories, the relatively new modified DLCN tools have not performed as expected, for either domestic Chinese populations or international populations (17, 18). For various heterozygous FH phenotypes, detection rates that use clinical measures and genetics cannot be significantly improved by simply raising the threshold of a single variable.

At present, prediction models based on datasets with a great number of variables and large sample sizes for FH risk assessment have been established in both the United States and Europe. For example, the FAMCAT in the United Kingdom (19), SEARCH (20), and FindFH algorithms (21) in the United States rely on the logistic regression models and have areas under the receiver operating curve (AUC) greater than 0.8. To further enhance model performance, machine learning (ML) models have been applied. The FindFH model has been established using random forest analysis and was found to have an improved AUC of 0.89 (21). Further ML models based on gradient boosting machines, neural networks, and ensemble learning have reached AUCs of greater than 0.89, which is a substantial improvement compared to the standard logistic model (AUC = 0.81) (22). Even though ML algorithms have enhanced model performance, the “black-box” feature has resulted in unknown correlations and distribution of each variable. Therefore, researchers in this field generally prefer logistic regression-based models to ML models. However, as interpretable ML methods emerge, the aforementioned situation will no longer exist. Indeed, interpreted ML methods have revealed non-linear correlations across different population samples, including biomethane production (23) and gut microbiome features in type 2 diabetes (24).

Additionally, hybridizing existing FH risk assessment tools could achieve higher clinical and genetic detection rates than unadjusted, single tools (25). This will also help establish tools for specific populations. Therefore, we aimed to assess the risk discovery performance of a hybrid model, which intercalated existing FH risk assessment tools with ML methods, based on a Chinese ASCVD patient sample.

## Materials and Methods

### Study Population

Data from 6,208 patients with ASCVD diagnosis at first discharge were collected from January 2012 to June 2020 in Peking Union Medical College Hospital (PUMCH). Data were then de-identified for anonymity. Cases with the following characteristics were excluded: (a) those without an LDL-c reading; (b) those with a history of ASCVD; (c) cases without total cholesterol (TC) record; and (d) those younger than 18 years, given the different criteria for the children.

**FIGURE 1:**
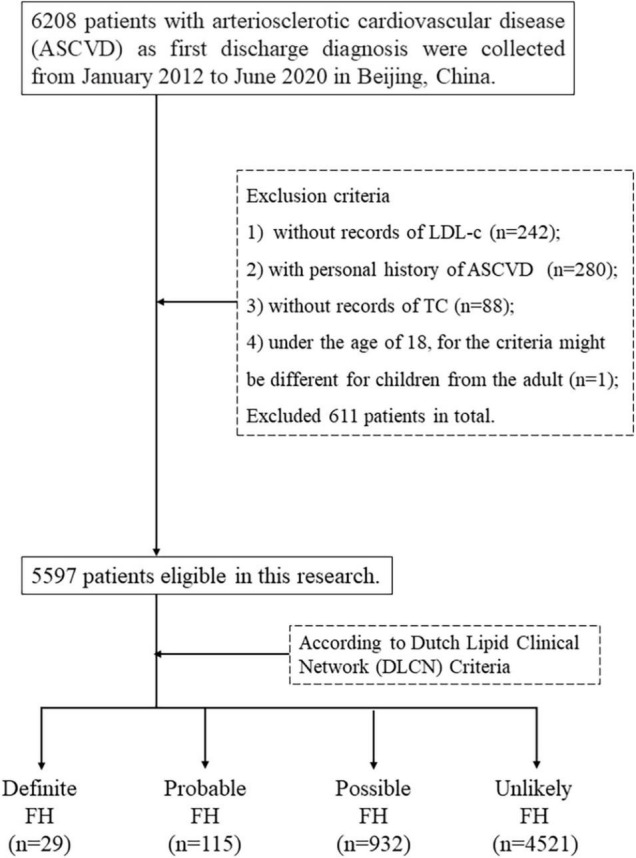
The exclusion process of first-ever ASCVD dataset.

In total, 5,597 cases made up the final dataset for criteria assessment and model development. Figure 1 gives further details of the eligibility process. In total, 5,597 patients with first-ever ASCVD were included, with an average age of 63.02 ± 11.44 years. Of which, 71.34% (*n* = 3,993) were men, and the average BMI was 25.46 ± 3.32 kg/m^2^. Only 0.11% (*n* = 6) had tendon xanthomata. Overall, 24.71% (*n* = 1,383) had been diagnosed as having premature coronary heart disease. We divided them into four groups according to the FH likelihood through the DLCN. The average age of four groups is decreased with the raising FH likelihood; for example, the patients with definite FH were 42.41 ± 9.19 years on average. More patients have been diagnosis with premature coronary atherosclerotic heart disease with a raising FH likelihood, and its prevalence in the definite FH patients is 100%. We have provided more information that is useful for describing our sample of 5,597 participants in Table 1.

**TABLE 1:** Clinical characteristics for familial hypercholesterolemia (FH) patients identified by DLCN.

Variables	Total participants (%)	DLCN				
		
		Unlikely FH (%)	Possible FH (%)	Probable FH (%)	Definte FH (%)	*P*-value
Number of participants	5,597	4,521	932	115	29	
Age (years)	63.02 ± 11.44	64.33 ± 10.8	58.55 ± 12.28	53.2 ± 10.78	42.41 ± 9.19	<0.001*
Gender/Male	3,993 (71.34)	3,239 (71.64)	658 (70.6)	73 (63.48)	23 (79.31)	0.185
Body mass index (kg/m2)	25.46 ± 3.32	25.38 ± 3.31	25.78 ± 3.27	26.01 ± 3.47	25.34 ± 3.96	0.002*
Tendon xanthomata/Yes	6 (0.11)	0 (0)	0 (0)	4 (3.48)	2 (6.9)	<0.001*
HDL-C (mmol/L)	0.98 ± 0.25	0.97 ± 0.25	1.01 ± 0.24	1.03 ± 0.24	1.02 ± 0.26	<0.001*
LDL-C (mmol/L)	2.45 ± 0.91	2.23 ± 0.7	3.23 ± 0.8	4.2 ± 1.02	6.32 ± 1.98	<0.001*
Lp(a) (mg/L)	177.79 ± 215.07	172.89 ± 212.57	194.33 ± 221.28	227.11 ± 245.52	214.97 ± 228.57	0.002*
TC (mmol/L)	4.17 ± 1.1	3.92 ± 0.92	5.01 ± 1	6.14 ± 1.2	7.57 ± 1.62	<0.001*
TG (mmol/L)	1.73 ± 1.63	1.7 ± 1.73	1.84 ± 1.06	2.14 ± 1.28	2.23 ± 1.46	0.001*
**Smoke**						<0.001*
Non-smoker	2,417 (43.18)	1,991 (44.04)	377 (40.45)	45 (39.13)	4 (13.79)	
Ex-smoker	1,322 (23.62)	1,099 (24.31)	191 (20.49)	27 (23.48)	5 (17.24)	
Current smoker	1,858 (33.2)	1,431 (31.65)	364 (39.06)	43 (37.39)	20 (68.97)	
**Drink**						0.03*
Non-drinker	3,437 (61.41)	2,799 (61.91)	558 (59.87)	69 (60)	11 (37.93)	
Ex-drinker	355 (6.34)	299 (6.61)	48 (5.15)	6 (5.22)	2 (6.9)	
Drinking habits	1,805 (32.25)	1,423 (31.48)	326 (34.98)	40 (34.78)	16 (55.17)	
**Personal history**						
Hyperlipemia/Yes	1,962 (35.05)	1,498 (33.13)	402 (43.13)	49 (42.61)	13 (44.83)	<0.001*
Diabetes/Yes	2,056 (36.73)	1,680 (37.16)	329 (35.3)	35 (30.43)	12 (41.38)	0.333
Hypertension/Yes	3,649 (65.2)	2,987 (66.07)	577 (61.91)	70 (60.87)	15 (51.72)	<0.001*
premature CHD/yes	1,383 (24.71)	863 (19.09)	407 (43.67)	84 (73.04)	29 (100)	<0.001*
Stroke/yes	628 (11.22)	494 (10.93)	119 (12.77)	15 (13.04)	0 (0)	0.083
**Family history**						
Family history of pCHD/Yes	70 (1.25)	28 (0.62)	30 (3.22)	10 (8.7)	2 (6.9)	<0.001*
Family history of Hyperlipemia/Yes	76 (1.36)	49 (1.08)	16 (1.72)	6 (5.22)	5 (17.24)	<0.001*
Family history of Stroke/Yes	709 (12.67)	562 (12.43)	130 (13.95)	15 (13.04)	2 (6.9)	0.475

The $\bar{x}\pm S\,D$ has displayed continuous variables. The frequency (percentage) has for the categorical variables, in which we only present the frequency of the patients with the feathers (also the “Yes” group). The α = 0.05 and * are for the variables with P values under 0.05. premature CHD, premature coronary atherosclerotic heart disease; LDL-C, highest LDL cholesterol during admission; TC, highest total cholesterol during admission; TG, highest triglyceride during admission.

Peking Union Medical College Hospital approved the study protocol, and all participants were provided with information regarding the objectives of the study and provided formal consent to participate.

### Hybrid Risk Assessment Tools

To establish an FH tool for the mainland Chinese population, we selected several tools to hybridize, using the following three steps:

(I) We select the most frequently used tools in both Chinese and international populations. These included 11 risk assessment tools that intercalated varied items and cutoff values. The heads of these items can be divided into five levels, namely, lipid levels, physical examination, family history, clinical history, and genetic test. We have provided each of the factors included in the 11 tools in Table 2. Details for specific items have also been provided in the Supplementary Material Table 1. Lipid levels and physical examination are the two most common factors. Genetic testing was included in only five of the 11 tools but was not considered essential in any.

**TABLE 2:** Head to head comparison among 11 risk assessment tools.

Head of the tools	SBR [1]	DLCN [2]	MEDPED [3]	JFHMC [4]	LDL-C/TC [5]	AHA [6]	SCCFH [7]	Lp(a)+DLCN [8]	mDLCN [9]	TW [10]	CHC [11]
Lipid levels	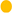	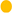	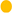	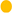	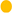	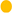	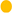	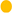	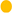	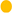	
Physical examination	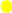	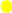		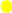		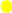	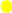	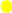		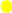	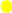
Family history	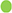	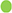	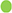	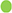		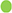	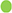	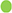	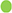	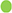	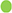
Clinical history		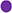		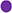		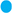	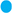		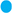	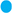	
Genetic test	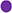	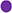				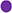		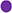		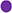	
Definite FH	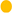 +( 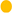 | 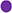 )	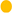 + 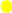 + 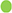 + 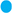 + 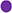 >8 points	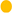 + 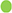	Homozygous FH: 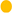 + 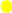 + 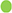 + 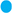 Heterozygous FH Any 2: 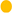 | 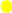 | 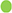	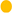	Homozygous FH: 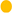 + 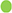 + 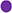 Heterozygous FH: 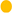 + 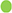 | 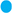 |	Any 2:1 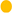 | 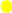 | 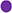	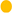 + 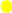 + 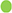 + 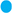 + 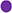 >6 points	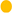 + 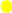 + 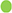 + 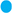 + 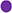 >8 points	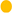 + 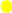 + 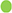 + 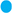 + 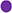 >8 points	Any 2:1 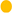 | 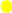 | 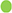

*SBR [1], Simon Broome Register; DLCN [2], Dutch Lipid Clinic network; MEDPED [3], make early diagnosis to prevent early deaths; JFHMC [4], Japanese FH Management Criteria; LDL-C/TC [5], TC&LDL-c; AHA [6], American Heart Association; Lp(a)DLCN [7], Lp(a)add DLCN; SCCFH [8], Simplified Chinese Criteria for Familial Hypercholesterolemia; mDLCN [9], modified DLCN for China; TW [10], Taiwan FH diagnostic criteria; CHC [11], 2018 Chinese criteria.*

(II) We also select candidate tools for the hybrid model. In order to assess the tool performance, the 11 tools were assessed using the DLCN as the reference. As a four-level criterion, the DLCN determines “probable” and “definite” patients into a high-risk group. The remainder was considered low-risk; two of the 10 risk assessment tools were selected for high sensitivity and specificity. Additionally, the definition of the three variables has to be mentioned before the assessment and include untreated LDL-c, BMI, and the cutoff of premature age. Data lipid-lowering treatments and duration of interventions were recorded. Lipid-lowering treatments mainly included statins, ezetimibe, niacin, and fibrates. Based on dosage, the treatments could be divided into three levels: high, median, and low potency. Evidence of receiving lipid-lowering interventions provides insights into LDL-c adjustments (26), which is necessary because most tools were assessed with previously untreated LDL-c patients. The weight and height were used to calculate body mass index (BMI), where necessary. Medical history was compared for the identification of premature cases which were based on two variables, namely, DLCN (with men < 55 years; women < 60 years), that is, premature coronary heart disease (pCHD), and the Taiwan criteria (27) which include men < 45 years and women < 55 years, that is, pCHDTW. Similarly, “premature” was defined using the DLCN, which stipulates that men younger than 55 years and women younger than 60 years, with a family history of premature coronary heart disease (pCHD_fh), or TW (men < 45 years; women < 55 years), known as the pCHD_fhTW. Both were included to determine which significantly correlated with our results.
(III) We hybridize the selected tools. We combined the two aforementioned tools with DLCN using a voting strategy (28). To discover high-risk patients, the hybrid result (HYR) has grouped “possible,” “probable,” and “definite” FH patients as high risk into a two-category outcome.

### Establish the Hybrid Familial Hypercholesterolemia Risk Assessment Tool With Machine Learning Algorithms

In order to establish the predictive model based on the dataset with a large number of variables and sample size, the ML models have been built as an FH risk predictor and to avoid personal biases during the multi-tool application, simultaneously. There were essentially three steps involved in establishing the predictive model.

#### Variable Selection

The first step in this process was variable selection. For this, 33 variables were collected directly through the electronic medical record (EMR) system. Variables were then divided into four categories namely demographics, patient histories, laboratory examinations, and family histories. Patient variables and family histories were based on the items from 11 frequently used risk assessment tools. Demographics, that is, age and gender and clinical characteristics including TC, LDL-c, HDL-c, and Lp(a) were included in respective laboratory indexes.

After stratifying the dataset with HYR into two subgroups, the variable selection process was considered under univariate analysis and through a four-variable selection method. The variable selection method included the Lasso (29), elastic net (30), random forest (RF) (31), and logistic regression. Variables used for the ML model setting were selected according to more than two of the four aforementioned methods.

#### Machine Learning Model for Familial Hypercholesterolemia Risk Prediction

The second step in this process was the FH risk predictor step which included establishing the Hybrid FH Risk Assessment Tool (HFHRAT). HYR was set as the outcome, with four ML methods taken as the main structure. These were then compared in terms of their performances using logistic models, including extreme gradient boosting (XGBoost) (32), RF (33), support vector machine (SVM) (34), and back-propagation artificial neural network (BPANN). We added Adaboost (35) and stacking (36) ensemble learning methods to balance data and model performance improvements. Adaboost is a homogeneous ensemble learning method that continuously samples from the dataset in order to construct a new model, based on the previous model performance.

Results were integrated using several models using the same ML method. Stacking is a heterogeneous method that links the results of base-level models set by different methods and further inputs into the meta-level model as independent variables. In this study, base-level models included RF, SVM, and BPANN, while simultaneously taking the logistic as the meta-level model. In total, six indexes assessed the performance of classifiers, including accuracy, sensitivity, precision–recall F measure (F), AUC, root-mean-squared error (RMSE), and G-mean value.

To simplify the use and explanation for participants, the probability model was also established using five indexes for performance assessment, consisting of AUC, RMSE, mean of calibration error (CAL mean) (37), and Brier scores (BS) (38). The best performing model in both classification and probability predictions was determined to be the final identifier. Due to the fact that ML models cannot generate probabilities during the classification process, an isotonic regression line (39) was added to recalibrate probabilities.

#### Correlations in the Hybrid Familial Hypercholesterolemia Risk Assessment Tool

**FIGURE 2:**
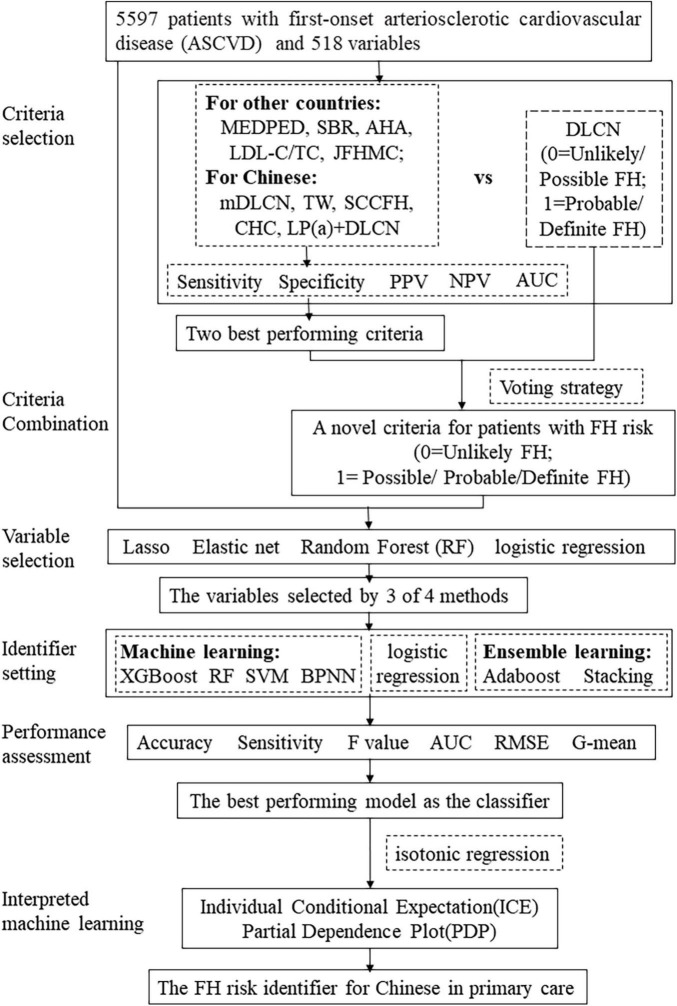
The roadmap of identifier setting.

During the final step, we interpreted the HFHRAT. ML models are not best suited to describing relations between independent and dependent variables. Therefore, we implemented interpretative machine learning methods, such as individual conditional expectation (ICE) and partial dependence plot (PDP), which are two model agnostic methods (40). The ICE and PDP were applied generally to provide explanations that were not bound to model settings (41). The ICE was used to display predictions for each sample through a single line, while the PDP was implemented to show how the marginal effect of one independent variable contrasted to the predicted value generated by the ML model through a fixed effect of the chosen variable and the average of other variables. The PDP line is always described as the average of the ICE. The whole process of setting identifiers has been presented as a schematic diagram in Figure 2.

To discover additional changes these hybrids present, we have compared all 33 variables for high-risk group predictions by five tools, including three existing tools, the HYR, and the HFHRAT. Continuous variables are presented as means with SDs. Categorical variables are presented as simple frequencies and percentages. Student’s *t*-test, the Kruskal–Wallis test, and χ^2^ analysis were implemented for inferential analysis.

Identifiers were constructed using R software (version 4.0.2). Refer to the “Supplementary Material” section for specifications and R packages used for imbalanced methods, machine learning, and interpretation methods.

## Results

### Eleven Existing Tool Selection and Hybrid

#### Tool Selection

Tools were assessed according to performance using the final dataset and were compared with the DLCN using five clinical measures. According to the DLCN assessment, the FH prevalence in our sample was 2.57%, which has two distinct elements (herein referred to as levels). In order to ensure consistency, each of the remaining assessment tools to be analyzed here was also modified to have two distinct levels. Additionally, without the result of the genetic test, the Japanese FH Management Criteria (JFHMC) could not predict the FH diagnosis and therefore was excluded because it cannot effectively identify high-risk cases.

The existing tools were selected in two aspects, namely sensitivity and specificity, to ensure the higher accuracy of the following hybrid tool. In terms of sensitivity, both the mDLCN and the LDL-C/TC tool have reached 97.22%, in which the latter assessed only laboratory test indexes, that is, LDL-C or TC, which yielded a high AUC (92.46%) and a slightly lower specificity (87.51%) compared to the mDLCN (AUC [95.06%] and specificity [92.90%]). In terms of specificity, TW and American Heart Association (AHA) reached 100%; however, the TW criteria had the highest sensitivity (41.54%) of the three. Therefore, the mDLCN and TW were the two selected tools. Table 3 gives more comprehensive summative data of the prevalence and the performances of the 10 different criteria compared with the DLCN.

**TABLE 3:** The prevalence and the performance of 10 different criteria compared with DLCN.

Criteria^a^	Level	Less-risk (%)	Risky (%)	Prevalence (%)	Sen (%)^d^	Spe (%)	PPV (%)	NPV (%)	AUC (%)
DLCN^b^	4	5453	144	2.57%					
mDLCN/risky	4	387	140	9.42%	97.22%	92.90%	26.57%	99.92%	95.06%
TW/risky	4	0	80	1.43%	55.56%	100%	100%	98.84%	77.78%
SCCFH/risky^c^	3	261	71	5.93%	49.31%	95.21%	21.39%	98.61%	72.26%
SBR/risky	3	18	23	0.73%	15.97%	99.67%	56.10%	97.82%	57.82%
AHA/risky	3	0	18	0.32%	12.50%	100%	100%	97.74%	56.25%
LDL-C/TC/risky	2	681	140	14.67%	97.22%	87.51%	17.05%	99.92%	92.37%
Lp(a)+DLCN/risky	2	140	91	4.13%	63.19%	97.43%	39.39%	99.01%	80.31%
MEDPED/risky	2	98	113	3.77%	78.47%	98.20%	53.55%	99.42%	88.34%
CHC/risky	2	8	14	0.39%	9.72%	99.85%	63.64%	97.67%	54.79%
JFHMC/risky	2	0	0	0	0	100%	100%	0	0

*^a^SBR, Simon Broome Register; DLCN, Dutch Lipid Clinic network; MEDPED, Make Early Diagnosis to Prevent Early Deaths; JFHMC, Japanese FH Management Criteria; LDL-C/TC, TC&LDL-c; AHA, American Heart Association; Lp(a)+DLCN, Lp(a)add DLCN; SCCFH, Simplified Chinese Criteria for Familial Hypercholesterolemia; mDLCN, modified DLCN for China; TW, Taiwan FH diagnostic criteria; CHC, 2018 Chinese criteria.*

*^b^According to the result of DLCN, the patients with probable and definite FH have been grouped as the risky FH group, while the rest of the patients were grouped in the less-risk FH group, so does it with mDLCN and TW.*

*^c^Among which SBR, AHA, and SCCFH are the three-level criteria, and grouped the patients in their highest two levels as the risky groups while comparing with DLCN in two-level, respectively. “Level” stands for the level of each criterion.*

*^d^We only present the results of the risky groups of the rest 10 criteria and assessed their performance with five indexes. In which, Sen is short for the sensitivity. Spe, specificity; PPV, the positive predictive value; NPV, negative predictive value; AUC, the area under the receiver operating characteristic curve.*

#### Tool Hybridization: Generating Hybrid Result

The Dutch Lipid Clinic Network, modified DLCN for China (mDLCN), and TW diagnostic criteria are three 4-level tools. With a voting strategy, their HYR was also a 4-level tool. Overall, 4,285 ASCVD patients were categorized as unlikely FH cases according to all three tools. However, different cutoff values for each tool led to an inconsistent risk-level assessment of a single sample, that is, a sample may be assessed by DLCN as probable FH, mDLCN as definite FH, and TW as possible FH. To discover all high-risk patients, we further grouped HYRs into two levels according to “possible,” “probable,” and “definite” FH cases as high risk, which means the aforementioned samples were identified as high-FH risk patients. For another group of samples, they were assessed by the DLCN as possible FH, mDLCN as probable FH, and TW as unlikely FH. According to the voting strategy, the results of the two tools owned to the high risk, and these samples were identified as the high-FH risk samples. For details of the voting strategy and the hybridizing process, see the Supplementary Material Table 2.

### Hybrid Familial Hypercholesterolemia Risk Assess Tool With Machine Learning Algorithms and Hybrid Result

The HYR modified the three existing tools into a robust single tool; however, the cutoff for every single item remained unknown using a crude voting strategy. When the HYP is applied, clinicians will have to learn several tools simultaneously, which may introduce personal biases into FH diagnosis. Therefore, the ML model was established based on the HYR, for better application and correlation clarification.

#### Variable Selection Based on Hybrid Result

The dataset was stratified according to HYR into high-risk and low-risk groups. In all, 33 significant variables remained between the groups for variable selection, while more than two variable selecting methods highlighted 14 candidate variables. LDL-c was identified as the most correlated variable by all methods, and the highest LDL-c during admission was included in the final model.

We included three variables identified in the DLCN and TW: history of premature CHD, premature stroke, and the family history of premature CHD. To avoid problems caused by overlapping information, we also eliminated variables with smaller coefficients. Finally, nine variables were left for further model development which coefficient in each variable selection method has been displayed in Table 4. The results of the 33 variables are given in Supplementary Material Table 3, and the top 20 variables of the variable selection result in Supplementary Material Table 4.

**TABLE 4:** The nine variables for machine learning (ML) model setting.

Variables	Logistic regression	RF	Elastic net	Lasso
LDL-c	5.71	569.48	0.2184	0.1991
pCHDTW	2.9	86.01	0.1588	0.1663
pCHD_fhTW	2.18	–	0.1422	0.1658
pStroke_fh	3.03	–	0.0961	0.1365
pStroke	7.37	–	0.3683	0.386
pPVD	8.63	–	0.1007	0.1338
Tendon xanthomas	23.83	–	0.2784	0.3512
Age	–	218.98	−0.0003	−0.0003
Lipid-low treat	4.47	540.8	–	0.1492

*For the result of the logistic regression, we identified the inclusion cutoff of logistic regression as 0.1 while the exclusion cutoff was 0.2. The Odd ratio and their 95% confidence interval have been displayed for each variable. RF stands for the random forest, and take the decreasing in Gini score for the variable selection. For Lasso and Elastic net, we have displayed the coefficients. LDL-c, the highest low density lipoprotein cholesterol during admission; pCHDTW, premature Coronary Heart Disease identified in Taiwan FH diagnostic criteria; pCHD_fhTW, Family History of premature Coronary Heart Disease identified in Taiwan FH diagnostic criteria; pStroke_fh, Family History of premature Stroke; pStroke, premature Stroke; pPVD, premature Peripheral Vascular Disease; Lipid-low treat, Lipid-lowering medication.*

#### Machine Learning Algorithms for Familial Hypercholesterolemia Risk Prediction

The HFHRAT was based on the aforementioned nine variables as the independent variables and the HYR as the dependent variable. The HYR divided the predictor setting dataset into two groups: 1,112 high-risk participants, that is, level 1, and 4,485 low-risk participants, that is, level 0 participants. The number of participants in the low-risk group was predictably four times larger than that in the high-risk group. We applied ensemble learning to handle problems caused by imbalanced data. Under the process of 10-fold cross-validation, 7-fold was randomly selected and combined to create a training dataset, while testing the model performance with the remaining 3-fold.

The HFHRAT is composed of the stacking models with the best performance in both classifier (AUC_class[94.85 ± 0.47]) and the probability predict model (AUC_prob[98.66 ± 0.27]). The BPANN also showed high AUC with AUC_class [90.65 ± 1.04] and AUC_prob [98.4 ± 0.27]), however, BPANN was not applied for further application, considering the lack of sensitivity [84.56 ± 2.19] and the predicted accuracy in the minority group can hardly be compromised. The XGboost model has shown high sensitivity [95.34 ± 1.52] but lacks separation in two classes, especially in the classifier (AUC_class [80.65 ± 1.25]; AUC_prob [92.96 ± 1.03]). The performance of classifiers and probability models is given in Table 5.

**TABLE 5:** The performance of familial hypercholesterolemia (FH) risk models.

Methods	Classifier						Probability model					
		
	Accuracy (%)	Sensitivity (%)	F	AUC_class (%)	RMSE_class	G score	AUC_prob (%)	RMSE_prob	Calmean	BS_1	BS_0	BS_ALL
XGBoost	71.79 ± 1.84	95.34 ± 1.52	0.5738 ± 0.0167	80.65 ± 1.25	0.5308 ± 0.0176	0.7928 ± 0.0141	92.96 ± 1.03	0.2787 ± 0.0274	0.0854 ± 0.0227	0.2962 ± 0.1276	0.0244 ± 0.0128	0.0784 ± 0.0163
RF	94.12 ± 0.52	86.09 ± 2.79	0.8532 ± 0.0137	91.1 ± 1.25	0.2423 ± 0.0106	0.9095 ± 0.0135	50 ± 0	0.4773 ± 0.0105	0.4606 ± 0.0122	0.2918 ± 0.021	0.2121 ± 0.0177	0.2279 ± 0.01
SVM	94.2 ± 0.45	82.82 ± 2.12	0.8503 ± 0.0121	89.93 ± 0.99	0.2406 ± 0.0094	0.8964 ± 0.0108	49.99 ± 0.03	0.3994 ± 0.0005	0.3025 ± 0.0007	0.6341 ± 0.0017	0.0418 ± 0.0006	0.1595 ± 0.0004
SVMBoost	94.28 ± 0.43	84.11 ± 2.49	0.8538 ± 0.0117	90.45 ± 1.1	0.2391 ± 0.0091	0.9022 ± 0.0119	49.98 ± 0.02	0.3994 ± 0.0004	0.3025 ± 0.0007	0.6341 ± 0.0017	0.0418 ± 0.0006	0.1596 ± 0.0003
BPANN	94.31 ± 0.48	84.56 ± 2.19	0.8552 ± 0.0126	90.65 ± 1.04	0.2384 ± 0.01	0.9043 ± 0.0112	98.4 ± 0.27	0.1972 ± 0.0076	0.0152 ± 0.0043	0.1168 ± 0.0143	0.0196 ± 0.0027	0.039 ± 0.003
BPANNBoost	94.38 ± 0.43	85.08 ± 2.12	0.8574 ± 0.0114	90.88 ± 0.98	0.237 ± 0.009	0.9069 ± 0.0105	50 ± 0.01	0.3999 ± 0.0026	0.3082 ± 0.0116	0.6193 ± 0.0287	0.0459 ± 0.0096	0.1599 ± 0.0021
LOG	93.77 ± 0.47	81.12 ± 2.25	0.838 ± 0.0132	89.01 ± 1.07	1.043 ± 0.0072	0.8865 ± 0.0118	87.42 ± 17.57	0.374 ± 0.2029	0.1909 ± 0.232	0.2797 ± 0.2448	0.1561 ± 0.2656	0.1807 ± 0.2012
LOGBoost	93.63 ± 0.65	82.18 ± 6.04	0.8361 ± 0.0207	89.32 ± 2.49	0.2522 ± 0.0128	0.8896 ± 0.0277	50 ± 0	0.4139 ± 0.0075	0.3635 ± 0.0195	0.4853 ± 0.0451	0.0935 ± 0.0187	0.1714 ± 0.0062
STACK	93.52 ± 0.47	97.06 ± 0.86	0.8564 ± 0.0092	94.85 ± 0.47	0.2543 ± 0.0092	0.9483 ± 0.0047	98.66 ± 0.27	0.2381 ± 0.0325	0.0683 ± 0.0299	0.2502 ± 0.103	0.01 ± 0.0079	0.0577 ± 0.0153

*XGBoost, eXtreme Gradient Boosting; RF, random forest; SVM, support vector machine; LOG, logistic regression; BPANN, BackPropagation Artificial Neural Network; BPANNBoost, an AdaBoost model settled with BPANN as the basic model; SVMBoost, an AdaBoost model settled with SVM as the basic model; LOGBoost, an AdaBoost model settled with logistic regression as the basic model; STACK, the stacking model with RF, SVM, BPANN, and LOG as the basic classifier and logistic regression as the meta classier. F, precision-recall F measure; AUC_class, area under the curve for classifier; AUC_prob, area under the curve for probability model; RMSE_class, root-mean-squared error of the classifier; RMSE_prob, root-mean-squared error of the probability model; Calmean, mean of calibration error; BS_1, Brier score for the samples with label 1; BS_0, Brier score for the samples with label 0; BS_ALL, Brier score for all samples.*

### The Interpretation of the Hybrid Familial Hypercholesterolemia Risk Assessment Tool and Efficiency Assessment for Tools

Partial dependence plot was used to explain correlations between age, LDL-c, and lipid-lowering therapies with FH risk. According to our dataset, 1,947 patients were yet to receive any lipid-lowering medication before admission. In total, 312, 3,210, and 128 patients had received low-, medium-, and high-potency statins before admission, respectively.

**FIGURE 3:**
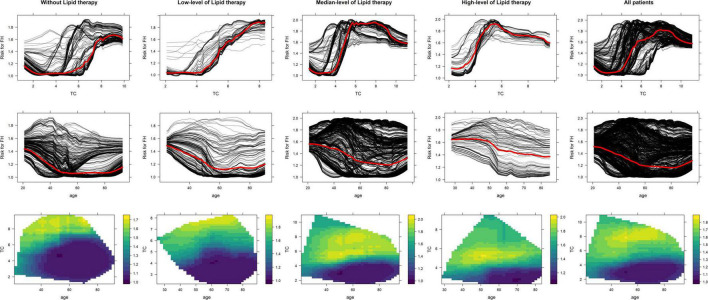
The interpretation among age, LDL-c, and lipid-lowering therapy to FH risk.

We divided the dataset into four subsets according to lipid-lowering therapies and analyzed correlations. According to PDP, the risk of FH correlated with an upward trend in all participants and across each subset. With the increasing level of lipid therapies, the risk of FH increased at the lower level with LDL-c. The decreasing trend of age to the FH risk was associated with limited changes among the different levels of therapies; however, the FH risk has been improved with the growing level of lipid-lowering therapy. The high-risk FH patients without lipid therapy were mostly patients aged <70 years and LDL-c > 4.5 mmol/L. As the level of lipid therapy increased, the range of the age and LDL-c have become more concentrated in the patients younger than 75 years with LDL-c > 4 mmol/L. See Figure 3 for PDPs.

To further discover the use of these hybrids in the tools, we have further compared the features of the high-risk groups predicted by five tools, including three existing tools, HYR, and the HFHRAT. Based on the voting strategy, HYR has found the median of three tools. With the ML algorithms, the HFHRAT has followed the data distribution and further adjusted the median position by enlarging the difference between the high- and low-risk groups. Of all 5,597 participants, 1,076 have been predicted to be high risk by DLCN, while 1,234 by the mDLCN, 959 by TW, 1,112 by HYR, and 1,116 by HFHRAT. For family history of hypertension, 39.6% of the high-risk participants defined by the DLCN have, while 39.7% in mDLCN, 39.1% in TW, 39.3% in HYR, and 40.1% in HFHRAT. Analytical results for the remaining features have been displayed in the Supplementary Material Table 3.

## Discussion

The hybrid FH risk assessment tool is a novel risk assessment tool based on hybridizing diagnostic with machine learning. This was designed for early identification of FH risk, specifically for mainland Chinese populations. The HFHRAT modified the median of the existing tools and avoided personal biases which can be a problem when applying hybridized tools. Despite having a rather heterogeneous population, this highlights a marked improvement and, of course, supports the need for a more accurate tool for mainland Chinese. It is hoped that this study will not only benefit those in China by identifying and preventing to development of ASCVD but will also help the global research community who are striving to improve risk assessment tools of this type.

### Prevalence of Familial Hypercholesterolemia

In our research, the overall prevalence of FH in patients with ASCVD (definite and probable) was 2.57%, of which definite FH accounted for 0.52% and probable FH was 2.05%, based on the DLCN. Although the FH prevalences are different between regions (42) and even between research (43), the prevalence of heterozygous FH mainly ranged from 1.17 [95% CI, 1.12–1.24] to 4.88 [95% CI, 4.17–8.33]) in the ASCVD (44). The estimation is higher than that of our research, 2.57%, for the range is based on the meta-analysis mostly consisting of the research for white individuals. Because of the different dietary habits, Chinese patients exhibit a lower level of TC and LDL-C than patients in Western counties (8). Such condition has slightly changed due to economic growth and increases in population cholesterol levels (5), although has not yet reached the level of TC and LDL-C in Western counties. The research with only Chinese patients has a prevalence similar to our study, in which the prevalence of probable and definite FH was 3.5% in patients undergoing coronary angiography (definite 1.0% and probable 2.5%) with coronary artery disease (CAD) (45) and 4.4% in patients with premature myocardial infarction (MI) (17). The prevalence may be different due to the constituent ratio of the study population by local and allopatry patients. The patients from specialty hospitals (in previous research studies) have more typical clinical symptoms than those in general hospitals (in our research). The prevalence estimated through phenotypic tools may elevate by such difference. The study population from a general hospital may own better consistency with the natural distribution of the FH patients in ASCVD. The consistency may attribute to the robustness of the model, while the improvement still needs further research to prove. Additionally, our participants are the ones with the first-ever ASCVD and exclude the ones with recurrence ASCVD. As the patient with FH is at a high risk of recurrent cardiovascular events (46), the prevalence may be downward by the exclusion.

### Hybrid Diagnosis Tool

The diagnosis of the study population originated from the hybrid result of the three existing tools. Each of them is applicable to diagnose both homozygous and heterozygous FH. Homozygous FH is an orphan disease, with a low prevalence of 1 in 300,000 individuals (47). Homozygous FH patients have very high LDL-C levels from birth, accelerated arterial stenosis and atherosclerosis, and premature death in their juvenile stage (48) due to myocardial infarction/acute coronary insufficiency. Our tools were built based on patients older than 18 years. Though a limited number of patients with homozygous FH may be, undeniable, include as well, the number shall be quite limited. According to the prevalence, the modeling individuals in our research are mainly composed of patients with heterozygous FH. Our tools are for heterozygote FH likelihood assessment. We are planning to recruit more homozygous FH specifically in our further research. For heterozygous FH diagnosis, the DLCN, SBR, and MEDPED perform reasonably well, although they do compromise a certain amount of either sensitivity or specificity. To satisfy the need for different regions, these tools have been modified in three ways: elevating or downgrading levels of existing items, adding new items, and assessing the importance of each item using new statistic algorithms. Most of the previous modifications are in the first category, for example, elevated the LDL-c level in DLCN for Canadians (49) and Chinese (15, 27), while LDL-c measures are superior for Italians (50). In the second category, lipoprotein(a) has long been discussed whether it should be included in the criteria (51). Lp(a)+DLCN (52) has put the statement into practice; however, it had very little effect on our participants. Logistic regression and machine learning methods (53) are the main algorithms involved in the third category. The FindFH (21) and the FH prediction model (FAMCAT) (54) are both logistic regression tools based on clinical records from US and UK patients, respectively. Both are improved based on random forest analysis (22). For the Chinese population, even the most popularly used DLCN has long been deemed unsuitable for Chinese patients (55), for the lipid level of Chinese was usually lower than that observed in Western societies. The discrepancy among the different tools in a new dataset mainly depended on the different cutoff levels in the premature onset or the lipid indexes (11). Assessing the FH risk in each participant with several different existing risk assessment tools and then considering their results comprehensively, the hybrid diagnosis tool could provide solutions to mitigate FH risk.

The 10 existing FH risk assessment tools we selected were from the former two categories and have estimated the FH risk in each participant in our dataset, which performed their consistency and difference with DLCN. In the consistency aspect, two modified DLCN criteria for Han Chinese specifically have left, including mDLCN and TW. The mDLCN has higher sensitivity than DLCN and the genetic test (40); however, low specificity has been its shortcoming. This mostly resulted from its modification in the cutoff of LDL-c, while the LDL-c ≥ 8.5 mmol/L matched the score 8 in the DLCN, but the same score in mDLCN matched the LDL-c ≥ 6 mmol/L. Therefore, the risk predicted using the mDLCN is higher than it ought to be for some. The TW criteria were also performed with high sensitivity in contrast to the genetic test when using the Han Chinese dataset (56). However, among the criteria with highest specificity (100%) based on our dataset, the TW has the highest sensitivity (41.54%), but this was less than that needed if the number of false negatives is reduced. Mostly resulted from a modification to the cutoff for premature development in participants and their first-level relatives, which as from <55 years old in men using the DLCN to <45 years old using the TW, and <60 to 55 years old for women. This constructive cutoff eliminated the high-risk participants without the early discovery of the high-lipid-leading disease. This means that at present, we cannot define a unified cutoff for mainland Chinese populations. We combined these tools with the voting strategy, with the result named HYR. The HYR has placed the median of the aforementioned three tools and improved the tool performance, although it brings more personal bias in the application and the exact cutoff value of each variable remains unknown.

### Establishing the Hybrid Familial Hypercholesterolemia Risk Assessment Tool

To eliminate the personal bias and get the cutoff value, the multi-variable models were settled as the classifier and probability predicting models with both the hybrid diagnosis and machine learning algorithms, named HFHRAT. The best performing model was finally set with the stacking ensemble algorithm, and nine variables yielded an AUC for the classifier, which was 94.85 ± 0.47%, and performed similarly to the previous ML models based on the participants from other regions (22), which ranged from 89 to 90%. All ML models performed better than the traditional logistic model.

For the number of the variables mentioned in the model, the FindFH model (21) for the U.S. population was a random forest model, with an AUC of 89% with 75 variables, while another random forest model (57) based on the same dataset has settled the model with 20 variables and reached an AUC of 94%. These three models also suggested that a large number of variables does not mean a high level of model performance. Using fewer variables for accurate predictions is easier and more likely to be accepted by clinicians. Our predicted model consisted of nine variables, in which relations with FH risk have been estimated by previous research, and mainly consisted of the items mentioned in the clinical guidelines. In addition to the dyslipidemia-related variables, personal and family histories (58) also affect the detection of patients at risk for FH. The tendon xanthomata (59) is an essential feature in FH diagnosis, although this is not often seen in patients from mainland China.

### Cutoff Values for Variables in the Hybrid Familial Hypercholesterolemia Risk Assessment Tool and Assessments

Most of the previous classifiers preferred the tree model for easier interpretation, which may compromise the accuracy of the model. The stacking ensemble learning model performed the best in our research, and its black box feature may be a disadvantage before the appearance of interpretation machine learning compared to the tree models. As a data-derived method, the correlations in the machine learning models mostly originated from the training dataset directly and can hardly acquire from the domain expertise, which brings potential pitfalls, such as unwanted confounding or interaction effects. The black box feature is the result of these effects. With two interpretation machine learning methods, PDP and ICE, the correlations in the model have been unveiled and assessed whether they corresponded with the natural laws. We have unveiled correlations among age, LDL-c, and the level of lipid-lowering therapy on the FH risk assessments. For the normal value of LDL-c ranging from 2.07 to 3.11 mmol/L, the participants with LDL-c ≥ 5.0 mmol/L can be defined as the possible FH by DLCN and TW, so as it is with the LDL-c ≥ 3.5 mmol/L in mDLCN. In HFHRAT, the LDL-c has been modified through the voting strategy and with machine learning methods using LDL-c levels in the three existing risk assessment tools, including DLCN, mDLCN, and the Taiwan FH diagnostic criteria (TW). LDL-c in the HFHRAT machine learning model highlighted the highest low-density lipoprotein cholesterol in first-ever ASCVD admissions. With the interpretation of the PDP, the participants with untreated LDL-c > 4.5 mmol/L without any lipid-lowering therapy are the possible FH, which is the median of the aforementioned three tools. The PDP and ICE have first estimated the LDL-c level of the participants with various levels of lipid-lowering therapies.

The univariate analysis provided in the Supplementary Material Table 3 provides the effect of the five tools. During the comparison among the five tools, the effect of the raw tools, the one with the hybrid diagnosis, and the one with both hybrid and ML algorithms, have been compared with each other. For the number of participants in the high-risk group, the HFHRAT consisted of 1,116 participants which are lower than that in the mDLCN (*n* = 1,234), higher than that in the DLCN (*n* = 1,076), TW (*n* = 959), and modified HYR (*n* = 1,112). The HYR has found the “median” position in these tools, while the HFHRAT has modified this “median” position based on the data distribution resulting from the features of the machine learning algorithms. For example, lp(a) is 199.21 ± 226.79 mg/L in the high-risk group of HYR, which is lower than that of the TW (201.52 ± 230.90 mg/L) but higher than that of the DLCN (198.39 ± 224.20 mg/L) and mDLCN (198.39 ± 225.05 mg/L). Although lp(a) is 198.04 ± 228.51 mg/L in the high-risk group of HFHRAT and the modified process has a lower lp(a) value, the high lp(a) still has a good effect on FH risk assessment (52). However, its effect may be covered up as a continuous variable (60), and its normal cutoff value has not yet been generally agreed upon, which could hardly be leveled into the categorical variable.

The hybrid FH risk assessment tool is a machine learning model based on the HYR, which is a majority testing result (also named voting strategy in biostatistics) of three tools, including DLCN, mDLCN, and TW. The performance of each tool could be tested by sensitivity and specificity. Majority testing avoids the trade-off and results in relatively high overall values for both test characteristics (61). In this case, HYR is more robust than DLCN. However, the majority of testing required more independent tools, which have been validated by several large population cohorts. Such need could hardly be satisfied, for the existing FH risk assessment tools for specific Chinese populations are limited and lack external validation. Therefore, the data-derived machine learning model, HFHRAT, has been built to adjust the sensitivity and specificity through the data distribution. For example, the average age of patients in level 1 was lower than in level 0. However, the average age of level 1 was defined according to the DLCN, which was 57.54 ± 12.42 years. This was the oldest average in all five tools (mDLCN 56.54 ± 12.18 years; TW 56.93 ± 13.41 years; HYR 56.91 ± 12.63 years; and HFHRAT 57.02 ± 12.70 years old). While compared with premature CHD prevalence, there were 48.3% (*n* = 520) with premature CHD in the level 1 group defined using DLCN [mDLCN 669 (54.2%), TW 437 (45.6%), HYR556 (50.0%), and HFHRAT 543 (48.7%)]. Level 1 defined using HFHRAT may be higher in age, but this approach compromised the prevalence of premature CHD and several other specific clinical features of patients with FH. However, this provided the unique potential for HFHRAT in FH likelihood detection and highlights its effectiveness in early diagnosis. Nevertheless, FH still requires results from patients at a longer follow-up and with genetic testing. Other variables with levels between 1 and 0 were divided according to the five risk assessment tools, provided in the Supplementary Material Table 3.

### Study Limitations

While there were some advantages and this study adds to the evidence base, there are few limitations to this study. First, 5,597 participants were all from a single center. This is likely to have skewed data to some extent, although further biases are introduced when analyzing multi-centric datasets. However, the final identifier (which is provided in the Supplementary Material) could be used to assess further datasets. Second, the results of genetic testing have not been included in this study. The HFHRAT aimed at identifying patients with a high FH likelihood and was therefore provided with advice around early genetic tests and lipid-lowering therapies. The participants identified as high risk required further confirmatory testing, which followed clinical guidelines. Third, the PDP made predictions for each sample according to others, which may further confound findings. Large-scale studies analyzing age-stratified groups might help overcome these biases.

In conclusion, the HFHRAT, for FH early diagnosis of the Chinese population, was set according to a stacking ensemble learning model based on the hybrid diagnosis (HYR), a voting strategy tool with three tools to avoid discrepancies among the DLCN, mDLCN, and TW. The mDLCN and TW were selected for consistency with the DLCN, which is most commonly used in clinical practice. The PDP and ICE revealed that the Chinese participants were younger than 75 years and untreated LDL-c > 4.5 mmol/L with the wFH risk. We would encourage these participants to assess their FH risk through the HFHRAT, and the high-risk ones shall further receive the genetic test in addition to the lipid-lowering therapy. We envisage that this risk assessment tool could perform efficiently in FH diagnosis in China and reduce the development of ASCVD and associated deaths.

## Data Availability Statement

The original contributions presented in this study are included in the article/Supplementary Material, further inquiries can be directed to the corresponding author.

## Ethics Statement

The studies involving human participants were reviewed and approved by Peking Union Medical College Hospital, Chinese Academy of Medical Sciences, Beijing, China. The patients/participants provided their written informed consent to participate in this study.

## Author Contributions

SZ, LW, and JG designed the work, while ZT and YJ provided valuable advice in terms of design and interpretation. LW and JG have collected and interpreted the dataset. LW created the codes and put them into practice. SS helped to write and edit this report. All authors contributed to the article and approved the submitted version.

## Conflict of Interest

The authors declare that the research was conducted in the absence of any commercial or financial relationships that could be construed as a potential conflict of interest.

## Publisher’s Note

All claims expressed in this article are solely those of the authors and do not necessarily represent those of their affiliated organizations, or those of the publisher, the editors and the reviewers. Any product that may be evaluated in this article, or claim that may be made by its manufacturer, is not guaranteed or endorsed by the publisher.

## Supplementary Material

The Supplementary Material for this article can be found online at: https://www.frontiersin.org/articles/10.3389/fcvm.2022.893986/full#supplementary-material and https://github.com/wangleiangel/Developing-a-hybrid-risk-assessment-tool-for-familial-hypercholesterolemia-A-machine-learning-study

## References

[1] Brown MS Goldstein JL. A receptor-mediated pathway for cholesterol homeostasis. Science. (1986) 232:34–47. 10.1126/science.3513311 3513311

[2] Florentin M Kostapanos MS Elisaf MS Liberopoulos EN. Prevalence, identification, and scouting for familial hypercholesterolaemia including registries. *Curr Pharm Des.* (2018) 24:3605–15. 10.2174/1381612824666181009103440 30306861

[3] Kalra S Chen Z Deerochanawong C Shyu KG Tan RS Tomlinson B Familial hypercholesterolemia in Asia pacific: a review of epidemiology, diagnosis, and management in the region. *J Atheroscler Thromb.* (2021) 28:417–34. 10.5551/jat.56762 33746137 PMC8193778

[4] Beheshti SO Madsen CM Varbo A Nordestgaard BG. Worldwide prevalence of familial hypercholesterolemia: meta-analyses of 11 million subjects. *J Am Coll Cardiol.* (2020) 75:2553–66. 10.1016/j.jacc.2020.03.057 32439005

[5] Tomlinson B Hu M Chow E. Current status of familial hypercholesterolemia in Chinese populations. *Curr Opin Lipidol.* (2019) 30:94–100. 10.1097/mol.0000000000000580 30649024

[6] Hu H Cheng J Lin S Wang S Chen X. Calcified Aortic valve disease in patients with familial hypercholesterolemia. *J Cardiovasc Pharmacol.* (2020) 76:506–13. 10.1097/fjc.0000000000000890 33165132

[7] McGowan MP Hosseini Dehkordi SH Moriarty PM Duell PB. Diagnosis and treatment of heterozygous familial hypercholesterolemia. *J Am Heart Assoc.* (2019) 8:e013225. 10.1161/jaha.119.013225 31838973 PMC6951065

[8] Chen P Chen X Zhang S. Current status of familial hypercholesterolemia in China: a need for patient FH registry systems. *Front Physiol.* (2019) 10:280. 10.3389/fphys.2019.00280 30949068 PMC6435575

[9] Civeira F. Guidelines for the diagnosis and management of heterozygous familial hypercholesterolemia. *Atherosclerosis.* (2004) 173:55–68. 10.1016/j.atherosclerosis.2003.11.010 15177124

[10] Thorogood M. Risk of fatal coronary heart disease in familial hypercholesterolaemia. Scientific steering committee on behalf of the simon broome register group. *BMJ.* (1991) 303:893–6. 10.1136/bmj.303.6807.893 1933004 PMC1671226

[11] Ibrahim S Reeskamp LF Stroes ESG Watts GF. Advances, gaps and opportunities in the detection of familial hypercholesterolemia: overview of current and future screening and detection methods. *Curr Opin Lipidol.* (2020) 31:347–55. 10.1097/mol.0000000000000714 33027222

[12] Watts GF Gidding SS Mata P Pang J Sullivan DR Yamashita S Familial hypercholesterolaemia: evolving knowledge for designing adaptive models of care. *Nat Rev Cardiol.* (2020) 17:360–77. 10.1038/s41569-019-0325-8 31974482

[13] Pang J Sullivan DR Brett T Kostner KM Hare DL Watts GF. Familial hypercholesterolaemia in 2020: a leading tier 1 genomic application. *Heart Lung Circ.* (2020) 29:619–33. 10.1016/j.hlc.2019.12.002 31974028

[14] Haralambos K Ashfield-Watt P McDowell IF. Diagnostic scoring for familial hypercholesterolaemia in practice. *Curr Opin Lipidol.* (2016) 27:367–74. 10.1097/mol.0000000000000325 27389632

[15] Shi Z Yuan B Zhao D Taylor AW Lin J Watts GF. Familial hypercholesterolemia in China: prevalence and evidence of underdetection and undertreatment in a community population. *Int J Cardiol.* (2014) 174:834–6. 10.1016/j.ijcard.2014.04.165 24801084

[16] Cao YX Sun D Liu HH Jin JL Li S Guo YL A novel modified system of simplified Chinese criteria for familial hypercholesterolemia (SCCFH). *Mol Diagn Ther.* (2019) 23:547–53. 10.1007/s40291-019-00405-1 31172370

[17] Cui Y Li S Zhang F Song J Lee C Wu M Prevalence of familial hypercholesterolemia in patients with premature myocardial infarction. *Clin Cardiol.* (2019) 42:385–90. 10.1002/clc.23154 30637778 PMC6712327

[18] Harada-Shiba M. How can we improve the diagnosis rate of familial hypercholesterolemia by amending diagnosis criteria? *Circ J.* (2021) 85:898–9. 10.1253/circj.CJ-21-0076 33762528

[19] Weng S Kai J Akyea R Qureshi N. Detection of familial hypercholesterolaemia: external validation of the FAMCAT clinical case-finding algorithm to identify patients in primary care. *Lancet Public Health.* (2019) 4:e256–64. 10.1016/s2468-2667(19)30061-1 31054643 PMC6506568

[20] Safarova MS Liu H Kullo IJ. Rapid identification of familial hypercholesterolemia from electronic health records: the SEARCH study. *J Clin Lipidol.* (2016) 10:1230–9. 10.1016/j.jacl.2016.08.001 27678441 PMC9229555

[21] Myers KD Knowles JW Staszak D Shapiro MD Howard W Yadava M Precision screening for familial hypercholesterolaemia: a machine learning study applied to electronic health encounter data. *Lancet Digit Health.* (2019) 1:e393–402. 10.1016/s2589-7500(19)30150-5 33323221 PMC8086528

[22] Akyea RK Qureshi N Kai J Weng SF. Performance and clinical utility of supervised machine-learning approaches in detecting familial hypercholesterolaemia in primary care. *NPJ Digit Med.* (2020) 3:142. 10.1038/s41746-020-00349-5 33145438 PMC7603302

[23] De Clercq D Wen Z Fei F Caicedo L Yuan K Shang R. Interpretable machine learning for predicting biomethane production in industrial-scale anaerobic co-digestion. *Sci Total Environ.* (2020) 712:134574. 10.1016/j.scitotenv.2019.134574 31931191

[24] Gou W Ling CW He Y Jiang Z Fu Y Xu F Interpretable Machine learning framework reveals robust gut microbiome features associated with type 2 diabetes. *Diabetes Care.* (2021) 44:358–66. 10.2337/dc20-1536 33288652 PMC7818326

[25] Eid WE Sapp EH Wendt A Lumpp A Miller C. Improving familial hypercholesterolemia diagnosis using an EMR-based hybrid diagnostic model. *J Clin Endocrinol Metab.* (2022) 107:1078–90. 10.1210/clinem/dgab873 34871430 PMC8947798

[26] Haralambos K Whatley SD Edwards R Gingell R Townsend D Ashfield-Watt P Clinical experience of scoring criteria for familial hypercholesterolaemia (FH) genetic testing in wales. *Atherosclerosis.* (2015) 240:190–6. 10.1016/j.atherosclerosis.2015.03.003 25797312

[27] Li YH Ueng KC Jeng JS Charng MJ Lin TH Chien KL 2017 Taiwan lipid guidelines for high risk patients. *J Formos Med Assoc.* (2017) 116:217–48. 10.1016/j.jfma.2016.11.013 28242176

[28] Giannakoulias S Shringari SR Liu C Phan HAT Barrett TM Ferrie JJ Rosetta machine learning models accurately classify positional effects of thioamides on proteolysis. *J Phys Chem B.* (2020) 124:8032–41. 10.1021/acs.jpcb.0c05981 32869996 PMC7759720

[29] Tibshirani R. Regression shrinkage and selection via the LASSO. *J Royal Statist Soc.* (1996) 73:273–82. 10.1111/j.1467-9868.2011.00771.x

[30] Zou H Hastie T. Regularization and variable selection via the elastic net. *J R Stat Soc.* (2005) 67:301–20. 10.1111/j.1467-9868.2005.00503.x

[31] Wang L Wang Y Chang Q. Feature selection methods for big data bioinformatics: a survey from the search perspective. *Methods.* (2016) 111:21–31. 10.1016/j.ymeth.2016.08.014 27592382

[32] Chen T Guestrin C. XGBoost: a scalable tree boosting system. In: *Proceedings of the 22nd ACM SIGKDD International Conference on Knowledge Discovery and Data Mining.* (San Francisco: CA: Association for Computing Machinery) (2016). p. 785–94.

[33] Breiman L. Random forests, machine learning 45. *J Clin Microbiol.* (2001) 2:199–228.

[34] Noble WS. What is a support vector machine? *Nat Biotechnol.* (2006) 24:1565–7. 10.1038/nbt1206-1565 17160063

[35] Schapire RE Freund Y Barlett P Lee WS. Boosting the margin: a new explanation for the effectiveness of voting methods. In: *Proceedings of the Fourteenth International Conference on Machine Learning.* (Burlington, MA: Morgan Kaufmann Publishers Inc) (1997). p. 322–30.

[36] Palanisamy S Kanmani S. Optimization of stacking ensemble configurations through artificial bee colony algorithm. *Swarm Evol Comput.* (2013) 12:24–32. 10.1016/j.swevo.2013.04.004

[37] Caruana R Niculescu-Mizil A Crew G Ksikes A. Ensemble selection from libraries of models. In: *Proceedings of the Twenty-First International Conference on Machine Learning.* Banff (2004). p. 18–25.

[38] Gw B. Verification of forecasts expressed of probability. *Mon Weather Rev.* (1950) 78:1–3.

[39] Niculescu-Mizil A Caruana R. Predicting good probabilities with supervised learning. In: *Proceedings of the 22nd International Conference on Machine Learning.* (New York, NY: ACM) (2005). p. 625–32.

[40] Ribeiro M Singh S Guestrin C. Model-agnostic interpretability of machine learning. *arXiv* [Preprint]. (2016). arXiv 1606.05386,

[41] Elshawi R Al-Mallah MH Sakr S. On the interpretability of machine learning-based model for predicting hypertension. *BMC Med Inform Decis Mak.* (2019) 19:146. 10.1186/s12911-019-0874-0 31357998 PMC6664803

[42] Toft-Nielsen F Emanuelsson F Benn M. Familial hypercholesterolemia prevalence among ethnicities-systematic review and meta-analysis. *Front Genet.* (2022) 13:840797. 10.3389/fgene.2022.840797 35186049 PMC8850281

[43] Vallejo-Vaz AJ Ray KK. Epidemiology of familial hypercholesterolaemia: community and clinical. *Atherosclerosis.* (2018) 277:289–97. 10.1016/j.atherosclerosis.2018.06.855 30270061

[44] Hu P Dharmayat KI Stevens CAT Sharabiani MTA Jones RS Watts GF Prevalence of familial hypercholesterolemia among the general population and patients with atherosclerotic cardiovascular disease: a systematic review and meta-analysis. *Circulation.* (2020) 141:1742–59. 10.1161/circulationaha.119.044795 32468833

[45] Li JJ Li S Zhu CG Wu NQ Zhang Y Guo YL Familial hypercholesterolemia phenotype in chinese patients undergoing coronary angiography. *Arterioscler Thromb Vasc Biol.* (2017) 37:570–9. 10.1161/atvbaha.116.308456 27932355

[46] Gencer B Nanchen D. Identifying familial hypercholesterolemia in acute coronary syndrome. *Curr Opin Lipidol.* (2016) 27:375–81. 10.1097/mol.0000000000000311 27092769

[47] Chacón-Camacho OF Pozo-Molina G Méndez-Catalá CF Reyes-Reali J Méndez-Cruz R Zenteno JC. Familial hypercholesterolemia: update and review. *Endocr Metab Immune Disord Drug Targets.* (2022) 22:198–211. 10.2174/1871530321666210208212148 33563162

[48] Cohen H Stefanutti C. Current approach to the diagnosis and treatment of heterozygote and homozygous FH children and adolescents. *Curr Atheroscler Rep.* (2021) 23:30. 10.1007/s11883-021-00926-3 33963467 PMC8105241

[49] Ruel I Brisson D Aljenedil S Awan Z Baass A Bélanger A Simplified canadian definition for familial hypercholesterolemia. *Can J Cardiol.* (2018) 34:1210–4. 10.1016/j.cjca.2018.05.015 30093300

[50] Noto D Spina R Giammanco A Barbagallo CM Ganci A Scrimali C Diagnosis of familial hypercholesterolemia in a large cohort of Italian genotyped hypercholesterolemic patients. *Atherosclerosis.* (2022) 347:63–7. 10.1016/j.atherosclerosis.2022.03.012 35339733

[51] Langsted A Nordestgaard BG. Lipoprotein(a) as part of the diagnosis of clinical familial hypercholesterolemia. *Curr Atheroscler Rep.* (2022) 24:289–96. 10.1007/s11883-022-01002-0 35107760

[52] Sun D Cao YX Li S Guo YL Wu NQ Gao Y A modified algorithm with lipoprotein(a) added for diagnosis of familial hypercholesterolemia. *Clin Cardiol.* (2019) 42:988–94. 10.1002/clc.23251 31436336 PMC6788465

[53] Pina A Helgadottir S Mancina RM Pavanello C Pirazzi C Montalcini T Virtual genetic diagnosis for familial hypercholesterolemia powered by machine learning. *Eur J Prev Cardiol.* (2020) 27:1639–46. 10.1177/2047487319898951 32019371

[54] Weng SF Kai J Andrew Neil H Humphries SE Qureshi N. Improving identification of familial hypercholesterolaemia in primary care: derivation and validation of the familial hypercholesterolaemia case ascertainment tool (FAMCAT). *Atherosclerosis.* (2015) 238:336–43. 10.1016/j.atherosclerosis.2014.12.034 25555265

[55] Kindt I Mata P Knowles JW. The role of registries and genetic databases in familial hypercholesterolemia. *Curr Opin Lipidol.* (2017) 28:152–60. 10.1097/mol.0000000000000398 28169870

[56] Migliara G Baccolini V Rosso A D’Andrea E Massimi A Villari P Familial hypercholesterolemia: a systematic review of guidelines on genetic testing and patient management. *Front Public Health.* (2017) 5:252. 10.3389/fpubh.2017.00252 28993804 PMC5622145

[57] Banda JM Sarraju A Abbasi F Parizo J Pariani M Ison H Finding missed cases of familial hypercholesterolemia in health systems using machine learning. *NPJ Digit Med.* (2019) 2:23. 10.1038/s41746-019-0101-5 31304370 PMC6550268

[58] Pereira AC. A roadmap for familial hypercholesterolaemia control. *Lancet Digit Health.* (2019) 1:e376–7. 10.1016/s2589-7500(19)30161-x 33323213

[59] Aljenedil S Ruel I Watters K Genest J. Severe xanthomatosis in heterozygous familial hypercholesterolemia. *J Clin Lipidol.* (2018) 12:872–7. 10.1016/j.jacl.2018.03.087 29778561

[60] Melita H Manolis AA Manolis TA Manolis AS. Lipoprotein(a) and cardiovascular disease: a missing link for premature atherosclerotic heart disease and/or residual risk. *J Cardiovasc Pharmacol.* (2022) 79:e18–35. 10.1097/fjc.0000000000001160 34694242

[61] Sonnenberg A. Combining the outcomes of endoscopy, laboratory testing, and professional judgement in gastroenterological decision-making. *Eur J Gastroenterol Hepatol.* (2017) 29:1321–6. 10.1097/meg.0000000000000974 29111998

