# Supplementary material for: Developing a Hybrid Risk Assessment Tool for Familial Hypercholesterolemia: A Machine Learning Study of Chinese Arteriosclerotic Cardiovascular Disease Patients
Source: Front Cardiovasc Med. 2022 Aug 3;9:893986. doi: 10.3389/fcvm.2022.893986 (PMC9381985; doi:10.3389/fcvm.2022.893986)
Supplement: Supplementary file 1 [file Data_Sheet_1.docx]

**Supplementary materials**

**Method**

According to Figure 2, the second step, the i**dentifier setting and explanation**, has three main steps, including variable selection, machine learning models setting and interpretation. Find below a brief description of each of these steps.

**1. Variable selection**

While useful and robust, prediction methods are limited to using a small number of predictors which operate in the same way and uniformly throughout their range. The features should not be with too large to avoid the over-fitting problems^[1]^. Therefore, three variable selection measures were applied in our research, including lasso, adaptive lasso and elastic net.

**1.1 Lasso**

Lasso^[2]^ is a shrinkage and selection method for linear regression. It connected soft-thresholding of wavelet coefficients, forward stage wise regression, and AdaBoost methods, while minimizing the usual sum of squared errors, with a bound on the sum of the absolute values of the coefficients. The lasso procedure is based on the “lars” R packages with the computation of the 10-fold cross-validated error curve.

**1.2 Adaptive lasso**

As a variant of lasso, adaptive lasso^[3]^ can enjoy the oracle property while the probability that it correctly selects the predictors with non-zero coefficients converges to one as the sample size increases and the estimators of the non-zero coefficients are asymptotically normal with the same mean and covariance that they would have if the zero coefficients were known in advance. It provides a different weights for each parameter in the penalty term:

.

In which the weights$\omega_{j}$ are the data-dependent variables. They are the inverse of the corresponding coefficients that are generated by the ridge regression. The “msgps” statement in “msgps” R package is applied to realize the procedure with the parameters penalty=“alasso”, gamma = 1, lambda = 0.

**1.3 Elastic net**

Elastic net[4]is a hybrid of ridge and lasso, has a penalty with both ridge and lasso parts.

It owns strengths in two aspects: firstly, it can produce more parsimonious models than ridge by performing variable selection. Secondly, it also tended to select or omit highly correlated predictors as a group. Such procedure is also realized by the “msgps” statement in msgps R package and set penalty as “enet” in addition.

**2. Machine learning**

**2.1 eXtreme Gradient Boosting(XGBoost)**

XGBoost popularized by Chen and Guestrin in 2016 [5], with Gradient boosting decision tree as the original model. XGBoost and Gradient boosting decision tree followed the same principle, and each new tree is created to reduce the residual of the previous model by the gradient boosting. Residual is designated by the differences between the actual and predicted values. Until the number of decision trees specify threshold, the model has been trained. Additionally, XGBoost optimizes the objective of function, size of the tree, and magnitude of the weights, which are controlled by standard regularization parameters. The better performing model has contributed by the hyperparameters. In which, gamma γ ∈ (0, +∞) denotes minimum loss reduction, which requires to make a split for making the partition on a leaf node of the tree. Minimum child weight w_mc_ ∈ (0, +∞) defines as minimum sum of instance weight, which means if the tree partition step results in a leaf node with the sum of instance weight less than w_mc_, then the tree will discard further partition. Early stop algorithm works for finding the optimal epoch number referring to given other hyperparameters. Finally, XGBoost also offered subsampling techniques and r_c_ ∈ (0, 1) column subsample ratio constructs in each tree. In the final step, grid search is used to regulate the hyperparameters in order to minimize the classification error.

**2.2 Random forest**

Random forest^[6]^is a bagging ensemble learning of tree model with a random vector to generate a bootstrap sample and take samples as training set of each decision tree. Then combine these tree models together and vote for a more popular model. The tree T in the forest $T=\{T_{i}\}$ consists of a root node, several internal nodes, several leaf nodes and edges connecting between these nodes. As a supervised learning method, the label of each sample is known and the aim of the method is to maximum the performance of the classification. To this aim, each node has to make a binary decision with such split function:

$$h_{\varphi}(v,\varphi_{i}):R^{d}\times\zeta\to\{0,1\}$$

In which $\varphi_{i}$is the split parameter. According to the$i^{th}$ node in the dataset, the split parameter $\zeta$and each node have two options, including 0 and 1.We set up the model through the “randomForest” R packages with the “randomForest” function and set the “mtry” parameter as 4.

**2.3 SVM**

The aim of the SVM^[7]^classifier is to build a bounder between different labels with the feather vectors from the training data$(x_{1},y_{1}),...,(x_{n},y_{n}),x_{i}\in R^{d}$and$y_{i}\in(-1,+1)$. In which x_i_ is the representation of the feather vector, $y_{i}$ is the label (-1 equals to negative,+1 equals to positive).The bounder is also called optimal hyper plane and can be defined as$wx^{T}+b=0$in which w is the weight vector, x is the feather vector of the training data and b is the bias.

Defined w and b with the following equation and find the maximum margin$1/\left\|w\right\|^{2}$.

$wx_{i}^{T}+b\geq+1$ if y_i_=1

$wx_{i}^{T}+b\leq\text{-}1$ if y_i_=-1

There are several methods to improve the prediction ability of the SVM model. For example change the kernel, tuning the parameters like the cost, gamma and so on. Our models are all based on Gaussian Radial Basis Function (RBF) kernel and set them with the “svm” statement in R package e1071 for SVM model with cost-sensitive calibration and “ksvm” statement in kernlab package for SVM model with SMOTE and imbalanced data.

**2.4 Back Propagation Artificial Neural Network**

Back Propagation Artificial Neural Network (BPANN)^[8]^ is a type of back propagation algorithms which is processed as a network composed with biological neural. While a training data is inputted into model under the direction “input layer—hidden layer—output layer”, the network will adjusting the weights and thresholds among neurons based on external information. The network we built with R package “nnet” has only one hidden layer in addition to input and output layer and set the parameter size=10, decay=0.01, maxit=400.

**2.5 Ensemble learning**

**2.5.1AdaBoost Algorithm**

AdaBoost algorithm^[9]^is an algorithm which combines several weak devices to solve the classification problems and predict the possibilities, so as to avoid the disadvantages of each classifier. We have applied the “train” statement in R package “caret” and set the method= “boost_all” in “trainControl” to realize the Adaboost function in each homogeneous ensemble model.

**2.5.2 Stacking algorithm**

Stacking^[10]^is also known as Stacked Generalization which combines the prediction of base-level classifiers by a meta-level classifier. The decisions made by the base-level classifiers are grouped up as a new database and a meta-level classifier will take the database as a training data for the final model setting. According to ZhiHua Zhou (2009)^[11]^, we’d like to select the best three single devices from above with the results in Table 5. After eliminate the extreme gradient boosting method (XGboost), the stacking model takes SVM, RF and logistic regression as the base-level learners while combine the results of them together and form a new dataset. Finally take logistic classifier as meta-level learner based on the new dataset above. The whole process is shown in Figure1.We have applied the “caretList” statement in R package “caret” to set the base-level classifiers. We set the parameter “methodList” as “c(“glm”, “rf”,“svmRadial”)” which stands for the SVM, RF and logistic regression are the base-level learners. And “caretStack” statement in R package “caretEnsemble” is used to defined the meta-level learner by setting the parameter “method” as “glm”. See Figure 2 for the structure of Stacking methods.

**3.** **Isotonic regression for Probability** **Recalibration**

To improve the practical of the model, we recalibrate the probability for the final identifier—the Stacking model. Though the dataset for current identifier setting were originated from a single center, the recalibration in probability will satisfied its further application to the novel dataset from multi-center, for more difference in probability prediction [12].

Isotonic regression has been applied for probability recalibration upon variety of machine learning algorithms, including  Naïve Bayes, SVM, and RF [13]. By generates a monotonically increasing mapping function to transform uncalibrated predictions to posterior probabilities, the classifier could accurately reflect true prevalence for each sample. The regression process shall following the two function below:

$$y_{i}=m(f_{i})+e_{i}$$

$$m={argmin}_{z}\sum{(y_{i}-z\left( f_{i} \right))}^{2}$$

The m is the main solution and is based on the mean squared error minimization, while y_i_ are the label of each sample, f_i_ are the raw results of the machine learning classifier, e_i_ are the error term [14].

**4. Interpreting Machine learning**

Once important features have been identified, it is often necessary to evaluate the relationship between them and the response variable. This can be achieved by constructing partial dependence plots (PDPs)[15], which visualize the relationship between a subset of model features (typically one to three features) and the target variable, while accounting for the average effect of other model predictors. The partial dependence of a target variable on input feature z_s_ can be defined as

$$f_{s}\left( z_{s} \right)=E_{z_{c}}\left[ \hat{f}\left( x \right) \right]=E_{z_{c}}\left[ \hat{f}\left( z_{s},z_{c} \right) \right]=\int\hat{f}\left( z_{s},z_{c} \right)p_{c}(z_{c})dz_{c}$$

where $x=\{x_{1},x_{2},\ldots,x_{p}\}$ represents predictors in a model, where the prediction function is$\hat{f}(x)$, x is partitioned into an interest set z_s_ and its compliment $z_{c}=x\{z_{s}$, p_c_ is the marginal probability density of z_c_ : $p_{c}\left( z_{c} \right)=\int p(x)dz_{s}$. The training dataset will estimate the above equation as: $\bar{f_{s}}\left( z_{s} \right)=\frac{1}{n}\sum_{i=1}^{n} \hat{f}(z_{s},z_{i,c})$where z_i,c_ (i=1,2,…,n) are values of z_c_ found in the training sample, meaning that effects of all the other features in the model are averaged out.

The partial dependence plot aims to visualize the average effect of a feature on the predicted risk of hypertension. Partial dependence is a global method as it does not focus on specific instances but on an overall average. ICE plot can be seen as the disaggregated view of PDP by displaying the estimated functional relationship for each instance in the dataset. The partial dependence plot can be seen as the average of the lines of an ICE plot[16]. In other words, ICE visualizes the dependence of the predicted risk of hypertension on particular features for each instance in the dataset. One main advantage of the ICE is that is easier to understand and more intuitive to interpret than the PDP. ICE suffers from the same disadvantage of PDP

**References**

1. Goldstein, B.A., A.M. Navar, and R.E. Carter, *Moving beyond regression techniques in cardiovascular risk prediction: applying machine learning to address analytic challenges.* European Heart Journal, 2016. **38**(23): p. 1805.

2. Tibshirani, R., *Regression Shrinkage and Selection via the Lasso.* Journal of the Royal Statistical Society: Series B Statistical Methodology, 1996. **73**(3): p. 273-282.

3. Zou, H., *The Adaptive Lasso and Its Oracle Properties.* Publications of the American Statistical Association, 2006. **101**(476): p. 1418-1429.

4. Zou H , H.T., *Addendum: Regularization and variable selection via the elastic net.* Journal of the Royal Statistical Society: Series B Statistical Methodology, 2005(67(5):768-768).

5. Chen, T. and C. Guestrin, *XGBoost: A Scalable Tree Boosting System.* ACM, 2016: p. 785–794.

6. Breiman, L., *Random forests, machine learning 45.* Journal of Clinical Microbiology, 2001. **2**: p. 199-228.

7. Noble, W.S., *What is a support vector machine?* Nature Biotechnology, 2006. **24**(12): p. 1565-1567.

8. Jesus, O.D. and M.T. Hagan, *Backpropagation Algorithms for a Broad Class of Dynamic Networks.* IEEE Transactions on Neural Networks, 2007. **18**(1): p. 14-27.

9. Schapire, R.E., et al. *Boosting the margin: A new explanation for the effectiveness of voting methods*. in *Fourteenth International Conference on Machine Learning*. 1997.

10. Shunmugapriya, P. and S. Kanmani, *Optimization of stacking ensemble configurations through Artificial Bee Colony algorithm.* Swarm & Evolutionary Computation, 2013. **12**(12): p. 24-32.

11. Zhou, Z.H., *Ensemble Learning*. 2009: Springer US. 125-142.

12. Todd and A. Alonzo, *Clinical Prediction Models: A Practical Approach to Development, Validation, and Updating: By Ewout W. Steyerberg.* Am.j.epidemiol, 2009.

13. Mervin, L., et al., *A Comparison of Scaling Methods to Obtain Calibrated Probabilities of Activity for Ligand-Target Predictions.* Journal of Chemical Information Modeling, 2020. **60**(10): p. 4546-4559.

14. Niculescu-Mizil, A. and R. Caruana. *Predicting good probabilities with supervised learning*. in *Machine Learning, Proceedings of the Twenty-Second International Conference (ICML 2005), Bonn, Germany, August 7-11, 2005*. 2005.

15. Friedman, J.H., *Greedy Function Approximation: A Gradient Boosting Machine.* Annals of Statistics, 2001. **29**(5): p. 1189-1232.

16. Alex, et al., *Peeking Inside the Black Box: Visualizing Statistical Learning With Plots of Individual Conditional Expectation.* Journal of Computational Graphical Statistics, 2015. **24**(1): p. 44-65.

**Supplementary materials Table 1. Items in each of the 11 criteria**

| Items(1) | SBR | DLCN | MEDPED | | JFHMC | LDL-C /TC | AHA | Lp(a)+DLCN | SCCFH | mDLCN | TW | CHC |
| --- | --- | --- | --- | --- | --- | --- | --- | --- | --- | --- | --- | --- |
| laboratory test | | | | | | | | | | | | |
| Numbers of item | 5 | 11 | 3 | | 6 | 2 | 8 | 7 | 5 | 7 | 13 | 3 |
| TC&  LDL-c | (a)pretreatment or highest on treatment TC> 7.5 mmol/L (290 mg/dl) in adults/ LDL-c > 4.9 mmol/L (190 mg/dL) in adults; TC> 6.7 mmol/L (260 mg/dl)/LDL-c> 4.0 mmol/L in children | (a)LDL – c ≥8.5 mmol/L (b)LDL-c 6.5–8.4 mmol/L (c)LDL- c 5.0–6.4 mmol/L (d)LDL – c 4.0–4.9 mmol/L | age group | TC/LDL-c(mmol/L) | (a)Before treatment TC ≥ 15.5 mmol/L) (b)Before treatment LDL-c: ≥ 4.7 mmol/L | TC>290mg/dL or 7.5mmol/L LDL-c>189mg/dL or 4.9mmol/L | (a)LDL-C >560 mg/dL (14 mmol/L) (b)LDL-C ≥400 mg/dL (10 mmol/L) (c)LDL-C ≥160 mg/dL (4 mmol/L) for children and ≥190 mg/dL (5 mmol/L) for adults | (a)Untreated LDL-C≥8.0 mmol/L (b)Untreated LDL-C6.0-8.0 mmol/L (c)Untreated LDL-C4.8-6.0 mmol/L (d)Lp(a)≥22 mg/dL | (a)LDL-C >=4.8mmol/L | (a)LDL-C ≥ 6 mmol/l (∼230 mg/dl) (b)LDL-C 5.0–5.9 mmol/l (190–224 mg/dl) (c)LDL-C 3.5–4.9 mmol/l (135–189 mg/dl) (d)LDL-C 2.5–3.4 mmol/l (97–134 mg/dl) 1 | (a)LDL-C≥330mg/dL（8.6mmol/l） (b)LDL-C 250-329mg/dL(6.5-8.6) (c)LDL-C 190-249mg/dL(5-6.5) (d)LDL-C 155-189mg/dL(3-5) | (a)Untreated LDL-C≥4.7 mmol/L(180mg/dL) |
|  |  |  | ＜20 | 7/5.2 |  |  |  |  |  |  |  |  |
|  |  |  | 20~29 | 7.5/5.7 |  |  |  |  |  |  |  |  |
|  |  |  | 30~39 | 8.8/6.2 |  |  |  |  |  |  |  |  |
|  |  |  | ＞40 | 9.3/6.7 |  |  |  |  |  |  |  |  |
| Physical Examination | | | | | | | | | | | | |
| tendon xanthomas | (b) in patient or relative (parent, child, sibling, grandparent, aunt, uncle) | (e)Tendon xanthomas  (f)Premature arcus |  | | (c)on the dorsal hands, elbows, and knees, or Achilles tendon thickening or nodular xanthoma on the skin |  | (d)xanthomata at <20 y of age | (e)Tendon xanthomas | (b)Tendon xanthomas |  | (e)Xanthoma (f)Corneal arcus (<45 y) | (b)Tendon xanthomas (<45 y) (c)Corneal arcus (<45 y) |
|  |  |  |  |  |  |  |  |  |  |  |  |  |

| Items | SBR | DLCN | MEDPED | | | | JFHMC | LDL-C/TC | AHA | Lp(a)+DLCN | SCCFH | mDLCN | TW | CHC |
| --- | --- | --- | --- | --- | --- | --- | --- | --- | --- | --- | --- | --- | --- | --- |
| Family history | | | | | | | | | | | | | | |
| myocardial infarction  (2) | (d)before age 50 in grandparent, aunt, uncle or before age 60 in parent, sibling or child. | (g)1st DR with premature (<55 years men;< 60 years women) coronary disease and vascular disease or LDL-c > 95th percentile | age group | 1st DR | 2nd DR | 3rd DR | (d)Parents’ family history of heterozygous FH. |  |  |  | (c)1st DR with premature(<55 years men;< 60 years women) coronary disease | (e)1st DR with premature (men: <55 years; women:<60 years) coronary artery disease or vascular disease. | (g)1st DR with early vascular/coronary disease (male <45 y, female <55 y) | (d)1st DR with FH OR early ASCVD, especially for coronary vascular disease |
| raised cholesterol | (e)in parent sibling or child, or level above 7.5 mmol/L (290 mg/dl) in grandparent, uncle, aunt | (h)1st DR with tendon xanthomata and/or arcuscornealis OR childhood (<18 years) with LDL-c > 95th percentile | ＜20 | 5.7/4 | 5.9/4.3 | 6.2/4.4 | (e)2nd DR FH or premature CAD (men <55, women <60 years) |  | (e)1st DR with LDL-C ≥160 mg/dL (4 mmol/L) for children and ≥190 mg/dL (5 mmol/L) for adults | (f)CHD or hypercholesterolemia | (d)1st DR with elevated LDL-C |  | (h)Adult 1st DR with LDL-C > 160 mg/dL (i)1st DR with xanthoma and/or corneal arcus (j)1st DR <18 y with LDL-C > 130 mg/dL |  |
|  |  |  | 20~29 | 6.2/4.4 | 6.5/4.7 | 6.7/4.8 |  |  |  |  |  |  |  |  |
|  |  |  | 30~39 | 7/4.9 | 7.2/5.2 | 7.9/5.4 |  |  |  |  |  |  |  |  |
|  |  |  | ＞40 | 7.5/5.3 | 7.8/5.6 | 8/5.8 |  |  |  |  |  |  |  |  |

| Items | SBR | DLCN | MEDPED | JFHMC | LDL-C/TC | AHA | Lp(a)+DLCN | SCCFH | mDLCN | TW | CHC |
| --- | --- | --- | --- | --- | --- | --- | --- | --- | --- | --- | --- |
| Clinical history | | | | | | | | | | | |
| premature CAD |  | (i)Patient with premature CAD (men <55, women <60 years) |  | (f)Premature CAD during childhood |  | (f)premature CAD | (g)Premature CHD |  | (f)Patient with premature (men: <55 years; women: <60 years) coronary artery disease | (k)Patient with early coronary artery disease (male <45 y, female <55 y) |  |
| premature cerebral or PVD |  | (j)Patient with premature cerebral or PVD(men <55, women<60 years) |  |  |  | (g)Patient with aortic valve disease |  |  | (g)Patient with premature (men: <55 years; women: <60 years) cerebrovascular or peripheral vascular disease | (l)Patient with early cerebral or peripheral arterial disease (male <45 y,female <55 y) |  |
| Functional | | | | | | | | | | | |
| Genetic test | (c) DNA-based evidence of an LDL receptor mutation or familial defective apo B-100 | (k)DNA mutations |  |  |  | (h)positive genetic testing for an LDL-C–raising gene defect (LDL receptor, ApoB, or PCSK9) |  | (e)DNA mutations |  | (m)Presence of functional mutation of LDL-R, ApoB-100, or PCSK9 gene |  |
| Score rules | (3) | (4) | (5) | (6) | (7) | (8) | (9) | (10) | (11) | (12) | (13) |

Notes, (1) SBR: Simon Broome Register; DLCN: Dutch Lipid Clinic network; MEDPED: Make Early Diagnosis to Prevent Early Deaths; JFHMC: Japanese FH Management Criteria; LDL-C/TC: TC&LDL-c; AHA : American Heart Association; Lp(a)DLCN: Lp(a)add DLCN; SCCFH: Simplified Chinese Criteria for Familial Hypercholesterolemia; mDLCN: modified DLCN for China; TW: Taiwan FH diagnostic criteria; CHC: 2018Chinese criteria;

(2) 1st DR: First degree relative; 2nd DR: Second degree relative; 3rd DR: Third degree relative;

(3) Definite FH:(a)&(b)|(c) Possible FH:(a)&(d)|(e)

(4) Score for each item(points):(a)8,(b)5,(c)3.(d)1,(e)6,(f)4,(g)1,(h)2,(i)2,(j)1,(k)8;By adding the scores together,Definite FH: > 8 points, Probable FH: 6–8 points, Possible FH: 3–5 points

(5) The paticipants and their first-, second- or third-degree relatives with higher serum cholesterol value, while comparaed to the cut-off one in the table through the age, were identified as FH

(6) Identified homozygous FH with:(a)&(c)&(d)&(f);Identified heterozygous FH with two or more of:(b),(c),(e);

(7) Identified the participants at high risk

(8) highly likely Homozygous FH:(a);(b)&(g)|(d) Homozygous FH:(b)&(e)&(h) Heterozygous FH:(c)&(e)|(f)|(h)

(9) Score for each item(points):(a)8,(b)4,(c)2,(d)1,(e)6,(f)1,(g)2;By adding the scores together,Definite FH: ≥6 points

(10) Definite FH:Meet any 2 out of 3 items,including (a),(b),(e) Probable FH:satisfied(a)&(c) or (a)&(d) Else are Hypercholesterolemia

(11) Score for each item(points):(a)8,(b)5,(c)3.(d)1,(e)1,(f)2,(g)1;By adding the scores together,Definite FH: > 8 points, Probable FH: 6–8 points, Possible FH: 3–5 points

(12) Score for each item(points):(a)8,(b)5,(c)3,(d)1,(e)6,(f)4,(g)1,(h)1,(i)2,(j)2,(k)2,(l)1,(m)8;By adding the scores together,Definite FH: > 8 points, Probable FH: 6–8 points, Possible FH: 3–5 points

(13) Meet any 2 out of 3 items(only one of the b and c can appeared in each identification).

**Supplementary materials Table 2. Voting process for the novel tool**

| DLCN | mDLCN | TW | Sample size | | HYR4level | HYR2level |
| --- | --- | --- | --- | --- | --- | --- |
| 1 | 1 | 1 | 4285 | 1 | | 0 |
| 1 | 2 | 1 | 130 | 1 | | 0 |
| 1 | 2 | 2 | 66 | 2 | | 1 |
| 1 | 1 | 2 | 40 | 1 | | 0 |
| 2 | 1 | 1 | 30 | 1 | | 0 |
| 2 | 1 | 2 | 4 | 2 | | 1 |
| 2 | 2 | 1 | 170 | 2 | | 1 |
| 2 | 2 | 2 | 341 | 2 | | 1 |
| 2 | 3 | 1 | 23 | >1 | | 1 |
| 2 | 3 | 2 | 326 | 2 | | 1 |
| 2 | 4 | 2 | 38 | 2 | | 1 |
| 3 | 1 | 3 | 4 | 3 | | 1 |
| 3 | 3 | 2 | 13 | 3 | | 1 |
| 3 | 3 | 3 | 21 | 3 | | 1 |
| 3 | 4 | 2 | 49 | ≥3 | | 1 |
| 3 | 4 | 3 | 28 | 3 | | 1 |
| 4 | 3 | 4 | 1 | 4 | | 1 |
| 4 | 4 | 2 | 2 | 4 | | 1 |
| 4 | 4 | 3 | 9 | 4 | | 1 |
| 4 | 4 | 4 | 17 | 4 | | 1 |

Notes, DLCN: Dutch Lipid Clinic network; mDLCN: modified DLCN for China; TW: Taiwan FH diagnostic tool; Sample size was the number of the samples in each subgroup. CFHRAT4level was the result of the voting strategy according to the three tools in which the subgroup of DLCN=3&mDLCN=4&TW=2 has further identified as the risky group for 2 of the 3 tools has leveled them in risky group(>=3). The same rule has applied to the subgroup of DLCN=2&mDLCN=3&TW=1, with the subgroup has final been leveled as unrisky group(>1). CFHRAT2level was the result of the novel tool. As a tool for primary care, the novel tool has grouped the unlikely FH(CFHRAT4level=1) as the unrisky FH group(CFHRAT2level=0), while the rest as the risky FH group(CFHRAT2level=1).

**Supplementary materials Table 3. Feathers of the FH risk group identified by four criteria**

| Variables | Total participants | | DLCN | | Statistic  (P-value) | mDLCN | | Statistic  (P-value) | TW | | Statistic  (P-value) | HYR | | Statistic  (P-value) | HFHRAT | | Statistic  (P-value) |
| --- | --- | --- | --- | --- | --- | --- | --- | --- | --- | --- | --- | --- | --- | --- | --- | --- | --- |
| levels |  | | 0(%) | 1(%) |  | 0(%) | 1(%) |  | 0(%) | 1(%) |  | 0(%) | 1(%) |  | 0(%) | 1(%) |  |
| Total sample size | 5597 | | 4521 | 1076 |  | 4363 | 1234 |  | 4638 | 959 |  | 4485 | 1112 |  | 4481 | 1116 |  |
| Age | 63.02±11.44 | | 64.33 ±10.80 | 57.54 ±12.42 | 17.98  (<0.001*) | 64.86 ±10.53 | 56.54 ±12.18 | 23.65  (<0.001*) | 64.28 ±10.56 | 56.93 ±13.41 | 18.66  (<0.001*) | 64.54 ±10.60 | 56.91 ±12.63 | 20.62  (<0.001*) | 64.52 ±10.59 | 57.02 ±12.70 | 20.30  (<0.001*) |
| Gender/Male | 3993(71.34) | | 3239  (71.6) | 754(70.1) | 1.05  (0.324) | 3118  (71.5) | 875(70.9) | 0.15  (0.729) | 3345  (72.1) | 648(67.6) | 8.05  (0.005*) | 3219  (71.8) | 774(69.6) | 2.05  (0.163) | 3212 (71.7) | 781 (70.0) | 1.26  (0.278) |
| Body mass index(kg/m2) | 25.46±3.32 | | 25.38  ±3.31 | 25.80  ±3.31 | -3.71  (<0.001*) | 25.36  ±3.30 | 25.82  ±3.37 | -4.31  (<0.001*) | 25.40  ±3.27 | 25.73  ±3.51 | -2.77  (0.006*) | 25.37  ±3.29 | 25.81  ±3.39 | -4  (<0.001*) | 25.34 ±3.30 | 25.92 ±3.33 | -5.22  (<0.001*) |
| Tendon xanthomata/Yes | 6(0.11) | | 0(0.0) | 6(0.6) | 25.24  (<0.001*) | 4(0.1) | 2(0.2) | 0.45  (0.861) | 0(0.0) | 6(0.6) | 29.05  (<0.001*) | 0(0.0) | 6(0.5) | 24.23  (<0.001*) | 1 (0.0) | 5 (0.4) | 15.12  (0.001*) |
| HDL-C(mmol/L) | 0.98±0.25 | | 0.97 ±0.25 | 1.01 ±0.24 | -4.11  (<0.001*) | 0.97 ±0.25 | 1.00 ±0.24 | -3.48  (0.001*) | 0.97 ±0.25 | 1.01 ±0.24 | -4.3  (<0.001*) | 0.97 ±0.25 | 1.01 ±0.24 | -3.97  (<0.001*) | 0.97 ±0.25 | 1.00 ±0.24 | -3.48  (0.001*) |
| Highest LDL cholesterol (LDL-C) (mmol/L) | 2.45±0.91 | | 2.23 ±0.70 | 3.41 ±1.04 | -44.88  (<0.001*) | 2.20 ±0.69 | 3.37 ±1.00 | -47.29  (<0.001*) | 2.23 ±0.71 | 3.52 ±1.03 | -46.76  (<0.001*) | 2.21 ±0.70 | 3.43 ±1.01 | -47.19  (<0.001*) | 2.21 ±0.69 | 3.45 ±0.99 | -39.80  (<0.001*) |
| Lp(a)( mg/L) | 177.79±215.05 | | 172.89 ±212.57 | 198.39 ±224.20 | -3.5  (<0.001*) | 171.97 ±211.83 | 198.39 ±225.05 | -3.82  (<0.001*) | 172.89 ±211.35 | 201.52 ±230.90 | -3.76  (<0.001*) | 172.48 ±211.76 | 199.21 ±226.79 | -3.71  (<0.001*) | 172.75 ±211.32 | 198.04 ±228.51 | -3.357  (<0.001*) |
| Highest total cholesterol (TC) (mmol/L) | 4.17±1.1 | | 3.92 ±0.92 | 5.20 ±1.17 | -38.7  (<0.001*) | 3.89 ±0.91 | 5.15 ±1.14 | -40.71  (<0.001*) | 3.93 ±0.92 | 5.31 ±1.15 | -40.48  (<0.001*) | 3.90 ±0.91 | 5.22 ±1.14 | -40.88  (<0.001*) | 3.90 ±0.91 | 5.24 ±1.12 | -37.18  (<0.001*) |
| Triglycerid (TG) (mmol/L) | 1.79±1.67 | | 1.70 ±1.73 | 1.88 ±1.11 | -3.41  (0.001*) | 1.68 ±1.73 | 1.92 ±1.15 | -4.6  (<0.001*) | 1.70 ±1.71 | 1.91 ±1.15 | -3.69  (<0.001*) | 1.69 ±1.72 | 1.92 ±1.15 | -4.27  (<0.001*) | 1.69 ±1.72 | 1.92 ±1.17 | -4.26  (<0.001*) |
| **Smoking status** | | | |  |  |  |  |  |  |  |  |  |  |  |  |  |  |
| Non-smoker | 2417(43.18) | | 1991  (44.0) | 426(39.6) | 25.6  (<0.001*) | 1938  (44.4) | 479(38.8) | 41.14  (<0.001) | 2013  (43.4) | 404(42.1) | 27.84  (<0.001*) | 1974  (44.0) | 443(39.8) | 28.42  (<0.001*) | 1979 (44.2) | 438 (39.2) | 30.616  (<0.001*) |
| Ex-smoker | 1322(23.62) | | 1099  (24.3) | 223(20.7) |  | 1070  (24.5) | 252(20.4) |  | 1146  (24.7) | 176(18.4) |  | 1096  (24.4) | 226(20.3) |  | 1092 (24.4) | 230 (20.6) |  |
| Current smoker | 1858(33.2) | | 1431  (31.7) | 427(39.7) |  | 1355  (31.1) | 503(40.8) |  | 1479  (31.9) | 379(39.5) |  | 1415  (31.5) | 443(39.8) |  | 1410 (31.5) | 448 (40.1) |  |
|  | | **Alcohol status** | |  |  |  |  |  |  |  |  |  |  |  |  |  |  |
| Non-drinker | 3437(61.41) | | 2799  (61.9) | 638(59.3) | 8.06  (0.018*) | 2708  (62.1) | 729(59.1) | 14.01  (0.001*) | 2841  (61.3) | 596(62.1) | 10.4  (0.006*) | 2769  (61.7) | 668(60.1) | 13.21  (0.001*) | 2765 (61.7) | 672 (60.2) | 11.03  (0.004*) |
| Ex-drinker | 355(6.34) | | 299(6.6) | 56(5.2) |  | 295  (6.8) | 60(4.9) |  | 316(6.8) | 39(4.1) |  | 306(6.8) | 49(4.4) |  | 304 (6.8) | 51 (4.6) |  |
| Drinking habits | 1805(32.25) | | 1423  (31.5) | 382(35.5) |  | 1360  (31.2) | 445(36.1) |  | 1481  (31.9) | 324(33.8) |  | 1410  (31.4) | 395(35.5) |  | 1412 (31.5) | 393(35.2) |  |
|  | | **Personal history** | |  |  |  |  |  |  |  |  |  |  |  |  |  |  |
| Hyperlipemia/Yes | 1962(35.05) | | 1498  (33.1) | 464(43.1) | 38.09  (<0.001*) | 1432  (32.8) | 530(42.9) | 43.34  (<0.001*) | 1555  (33.5) | 407(42.4) | 27.73  (<0.001*) | 1479  (33.0) | 483(43.4) | 42.81  (<0.001*) | 1481 (33.1) | 481(43.1) | 39.64  (<0.001*) |
| Hypertension/Yes | 3649(65.2) | | 2987  (66.1) | 662(61.5) | 7.91  (0.005*) | 2906  (66.6) | 743(60.2) | 17.34  (<0.001*) | 3074  (66.3) | 575(60.0) | 13.99  (<0.001*) | 2978  (66.4) | 671(60.3) | 14.41  (<0.001*) | 2966 (66.2) | 683(61.2) | 39.64  (0.002*) |
| Diabetes/Yes | 2056(36.73) | | 1680  (37.2) | 376(34.9) | 1.84  (0.187) | 1654  (37.9) | 402(32.6) | 11.77  (0.001*) | 1754  (37.8) | 302(31.5) | 13.69  (<0.001*) | 1687  (37.6) | 369(33.2) | 7.53  (0.007*) | 1686 (37.6) | 370 (33.2) | 7.69  (0.006*) |
| Stroke  /Yes | 628(11.22) | | 494(10.9) | 134(12.5) | 2.03  (0.17) | 514(11.8) | 114(9.2) | 6.24  (0.014*) | 542(11.7) | 86(9.0) | 5.9  (0.018*) | 513(11.4) | 115(10.3) | 1.08  (0.325) | 507 (11.3) | 121 (10.8) | 0.2  (0.693) |
| AF/Yes | 90(1.61) | | 81(1.8) | 9(0.8) | 5.01  (0.035*) | 81(1.9) | 9(0.7) | 7.73  (0.008*) | 84(1.8) | 6(0.6) | 7.06  (0.012*) | 81(1.8) | 9(0.8) | 5.6  (0.026*) | 80 (1.8) | 10 (0.9) | 4.47  (0.048*) |
| PVD/Yes | 125(2.23) | | 99(2.2) | 26(2.4) | 0.2  (0.736) | 101(2.3) | 24(1.9) | 0.6  (0.504) | 103(2.2) | 22(2.3) | 0.02  (0.984) | 102(2.3) | 23(2.1) | 0.17  (0.762) | 101 (2.3) | 24 (2.2) | 0.009  (0.924) |
| premature CHD/Yes | 1383(24.71) | | 863(19.1) | 520(48.3) | 399.39  (<0.001*) | 714(16.4) | 669(54.2) | 740.71  (<0.001*) | 946(20.4) | 437(45.6) | 270.65  (<0.001*) | 827(18.4) | 556(50.0) | 477.09  (<0.001*) | 840 (18.7) | 543(48.7) | 429.65  (<0.001*) |
| premature CHD TW/Yes | 473(8.45) | | 278(6.1) | 195(18.1) | 161.06  (<0.001*) | 220(5.0) | 253(20.5) | 297.17  (<0.001*) | 180(3.9) | 293(30.6) | 730.69  (<0.001*) | 216(4.8) | 257(23.1) | 385.51  (<0.001*) | 211 (4.7) | 262(23.5) | 406.778  (<0.001*) |
| premature PVD/Yes | 10(0.18) | | 0(0.0) | 10(0.9) | 42.09  (<0.001*) | 3(0.1) | 7(0.6) | 13.4  (0.001*) | 4(0.1) | 6(0.6) | 12.97(0.001*) | 3(0.1) | 7(0.6) | 15.81  (<0.001*) | 4 (0.1) | 6 (0.5) | 7.71  (0.005*) |
| premature PVD TW/Yes | 2(0.04) | | 0(0.0) | 2(0.2) | 8.41  (0.045*) | 0(0.0) | 2(0.2) | 7.07  (0.071) | 0(0.0) | 2(0.2) | 9.68  (0.03*) | 0(0.0) | 2(0.2) | 8.07  (0.051) | 1 (0.0) | 1 (0.1) | 1.13  (0.858) |
| premature Stroke/Yes | 60(1.07) | | 0(0.0) | 60(5.6) | 254.83  (<0.001*) | 22(0.5) | 38(3.1) | 60.15  (<0.001*) | 44(0.9) | 16(1.7) | 3.88  (0.072) | 19(0.4) | 41(3.7) | 89.48  (<0.001*) | 20 (0.4) | 40 (3.6) | 82.96  (<0.001*) |
| premature Stroke TW/Yes | 11(0.2) | | 0(0.0) | 11(1.0) | 46.31  (<0.001*) | 3(0.1) | 8(0.6) | 16.47  (<0.001*) | 0(0.0) | 11(1.1) | 53.3  (<0.001*) | 0(0.0) | 11(1.0) | 44.45  (<0.001*) | 0 (0.0) | 11 (1.0) | 44.26  (<0.001*) |
|  | | **Family history** | |  |  |  |  |  |  |  |  |  |  |  |  |  |  |
| Family history of pCHD/Yes | 70(1.25) | | 28(0.6) | 42(3.9) | 75.9  (<0.001*) | 27(0.6) | 43(3.5) | 63.97  (<0.001*) | 48(1.0) | 22(2.3) | 10.2  (0.002*) | 35(0.8) | 35(3.1) | 40.43  (<0.001*) | 38 (0.8) | 32 (2.9) | 29.5  (<0.001*) |
| Family history of pCHD TW/Yes | 25(0.45) | | 10(0.2) | 15(1.4) | 26.89  (<0.001*) | 7(0.2) | 18(1.5) | 36.46  (<0.001*) | 18(0.4) | 7(0.7) | 2.09  (0.238) | 11(0.2) | 14(1.3) | 20.59  (<0.001*) | 9 (0.2) | 16 (1.4) | 30.54  (<0.001*) |
| Family history of CHD/Yes | 1844(32.95) | | 1400  (31.0) | 444(41.3) | 41.72  (<0.001*) | 1335  (30.6) | 509(41.2) | 49.39  (<0.001*) | 1459  (31.5) | 385(40.1) | 27.16  (<0.001*) | 1388  (30.9) | 456(41.0) | 40.82  (<0.001*) | 1395 (31.1) | 449 (40.2) | 33.50  (<0.001*) |
| Family history of Hyperlipemia/Yes | 76(1.36) | | 49(1.1) | 27(2.5) | 13.19  (<0.001*) | 45(1.0) | 31(2.5) | 15.75  (<0.001*) | 48(1.0) | 28(2.9) | 21.08  (<0.001*) | 46(1.0) | 30(2.7) | 18.6  (<0.001*) | 47 (1.0) | 29 (2.6) | 16.02  (<0.001*) |
| Family history of Stroke/Yes | 709(12.67) | | 562(12.4) | 147(13.7) | 1.19  (0.298) | 543(12.4) | 166(13.5) | 0.88  (0.373) | 584(12.6) | 125(13.0) | 0.14  (0.747) | 562(12.5) | 147(13.2) | 0.38  (0.57) | 555 (12.4) | 154 (13.8) | 1.61  (0.222) |
| Family history of Hypertension/Yes | 1859(33.21) | | 1433  (31.7) | 426(39.6) | 24.42  (<0.001*) | 1369  (31.4) | 490(39.7) | 30.1  (<0.001*) | 1484  (32.0) | 375(39.1) | 18.09  (<0.001*) | 1422  (31.7) | 437(39.3) | 23.16  (<0.001*) | 1411 (31.5) | 448 (40.1) | 30.17  (<0.001*) |
| Family history of Diabetes/Yes | 937(16.74) | | 723(16.0) | 214(19.9) | 9.47  (0.002*) | 692(15.9) | 245(19.9) | 11.01  (0.001*) | 758(16.3) | 179(18.7) | 3.07  (0.088) | 718(16.0) | 219(19.7) | 8.68  (0.004*) | 716(16.0) | 221(19.8) | 9.38  (0.003*) |
| Family history of premature Stroke/Yes | 20(0.36) | | 10(0.2) | 10(0.9) | 12.24  (0.001*) | 7(0.2) | 13(1.1) | 21.55  (<0.001*) | 14(0.3) | 6(0.6) | 2.34  (0.218) | 11(0.2) | 9(0.8) | 7.96  (0.011*) | 12 (0.3) | 8 (0.7) | 3.88  (0.049*) |
| Family history of AF/Yes | 28(0.5) | | 22(0.5) | 6(0.6) | 0.09  (0.955) | 23(0.5) | 5(0.4) | 0.29  (0.758) | 25(0.5) | 3(0.3) | 0.82  (0.514) | 23(0.5) | 5(0.4) | 0.07  (0.976) | 23 (0.5) | 5 (0.4) | 0.002  (0.969) |
|  | | **Lipid-lowering medication** | | |  |  |  |  |  |  |  |  |  |  |  |  |  |
| Non lipid treatment | 1947(34.79) | | 1851  (40.9) | 96(8.9) | 494.68  (<0.001*) | 1807  (41.4) | 140(11.3) | 490.19  (<0.001*) | 1859  (40.1) | 88(9.2) | 423.7  (<0.001*) | 1849  (41.2) | 98(8.8) | 511.59  (<0.001*) | 1856 (41.4) | 91 (8.2) | 534.28  (<0.001*) |
| Low potency statin | 312(5.57) | | 269(6.0) | 43(4.0) |  | 262(6.0) | 50(4.1) |  | 270(5.8) | 42(4.4) |  | 264(5.9) | 48(4.3) |  | 266 (5.9) | 46 (4.1) |  |
| Medium potency statin | 3210(57.35) | | 2344  (51.8) | 866(80.5) |  | 2244  (51.4) | 966(78.3) |  | 2446  (52.7) | 764(79.7) |  | 2317  (51.7) | 893(80.3) |  | 2303 (51.4) | 907(81.3) |  |
| High potency statin | 128(2.29) | | 57(1.3) | 71(6.6) |  | 50(1.1) | 78(6.3) |  | 63(1.4) | 65(6.8) |  | 55(1.2) | 73(6.6) |  | 56 (1.2) | 72 (6.5) |  |

Notes, level 1 has included the patients with definite, probable, and possible FH, while the unlikely FH patients have been grouped into level 0, based on each criterion above. HYR is the combination of the DLCN, mDLCN, and TW with voting strategy and the combining process has been displayed in Supplementary materials Table 2. The $\bar{x}\pm SD$ has displayed continuous variables. The frequency(percentage) has for the categorical variables, in which we only present the frequency of the patients with the feathers(also the “Yes” group). The α=0.05 and * are for the feathers with P values under 0.05. “TW” after the feature represents the feather was identified by the TW criteria, as they were earlier in premature age definition. pCHD: premature CHD.

**Supplementary materials Table 4. Top 20 variables selected for the identifier**

| Variables | Logistic regression | RF | Elastic net | Lasso |
| --- | --- | --- | --- | --- |
| LDL-c | 5.71 | 569.48 | 0.2184 | 0.1991 |
| pCHDTW | 2.9 | 86.01 | 0.1588 | 0.1663 |
| pCHD | 3.25 | 240.48 | 0.1342 | 0.1356 |
| pCHD_fhTW | 2.18 | - | 0.1422 | 0.1658 |
| pStroke_fh | 3.03 | - | 0.0961 | 0.1365 |
| pCHD_fh | 3.25 | - | 0.1017 | 0.1166 |
| pStroke | 7.37 | - | 0.3683 | 0.386 |
| pPVD | 8.63 | - | 0.1007 | 0.1338 |
| pStrokeTW | 17.27 | - | 0.0472 | 0.0845 |
| tendon xanthomas | 23.83 | - | 0.2784 | 0.3512 |
| age | - | 218.98 | -0.0003 | -0.0003 |
| TC | - | 387.85 | 0.0199 | 0.0419 |
| Lipid-low treat | 4.47 | 540.8 | - | 0.1492 |
| BMI | -0.04 | - | - | 0.0005 |
| Hypertension_fh | 0.3 | - | - | 0.0039 |
| HDL-c | - | - | -0.0182 | -0.0514 |
| TG | - | - | -0.0026 | -0.01 |
| pPVDTW | - | - | 0.1607 | 0.259 |
| AF | - | - | - | 0.0259 |
| PVD | - | - | - | 0.0081 |

Notes, for the result of the logistic regression, we identified the inclusion cutoff of logistic regression as 0.1 while the exclusion cutoff was 0.2. The Odd ratio and their 95% confidence interval have been displayed for each variable. RF stands for the random forest, and take the decreasing in Gini score for the variable selection. For Lasso and Elastic net, we have displayed the coefficients. LDL-c: The highest low density lipoprotein cholesterol during admission; pCHDTW: premature Coronary Heart Disease identified in Taiwan FH diagnostic criteria; pCHD: premature Coronary Heart Disease; pCHD_fhTW: Family History of premature Coronary Heart Disease identified in Taiwan FH diagnostic criteria; pStroke_fh: Family History of premature Stroke; pCHD_fh: Family History of premature Coronary Heart Disease; pStroke: premature Stroke; pPVD: premature Peripheral Vascular Disease; pStrokeTW: premature Stroke identified in Taiwan FH diagnostic criteria; TC: The Highest total cholesterol during admission; Lipid-low treat: Lipid-lowering medication; BMI: Body mass index; Hypertension_fh: Family History of Hypertension; HDL-c: The highest high density lipoprotein cholesterol during admission; TG: The highest Triglycerid during admission; pPVDTW: premature Peripheral Vascular Disease identified in Taiwan FH diagnostic criteria; AF:[atrial](javascript:;) [fibrillation](javascript:;); PVD: Peripheral Vascular Disease.

**Supplementary materials Table 5. Parameters and R packages of models**

| Usage | Methods | R packages | Statement | Parameter Values |
| --- | --- | --- | --- | --- |
| Variable selection | Lasso | lars | lars | type="lar" |
|  | Adaptive Lasso | msgps | msgps | penalty=“alasso”, gamma = 1, lambda = 0 |
|  | Elastic net | msgps | msgps | penalty=“alasso”, gamma = 1, lambda = 0 |
| Machine learning&  Ensemble learning | SVM | e1071 | svm | type = "C-classification",kernel = "linear", cross = 0; in which cost and gamma are originated from tuning process.gamma is tuning in three values(0.5,1.0,2.0),cost is in (4,8,16) |
|  | Random forest | randomForest | randomForest | importance=TRUE, proximity=TRUE,mtry=4 |
|  | Logistic |  | “glm” and “step” | family=binomial |
|  | Adaboost | caret | train | trControl=trainControl(method = "boot_all", number = 10) |
|  | Stacking | caretEnsemble | “caretList”And “caretStack” | classProbs=TRUE, the classifiers in base level are set as “methodList=c("nnet", "rf","svmRadial")”;the model in meta-level can set the parameter “ method="glm"". |
|  | Weight vote |  | Set vote function | Weight=Accuracy of each model, limit=0.5(take possibility equal and over 0.5 patients as 1,else as 0) |
|  | Average vote |  | Set vote function | Limit=2(take possibility equal and over 2 patients as 1,else as 0) |
|  | extreme gradient boosting(XGBoost) | xgboost | xgboost | max_depth = 2, eta = 1, nthread = 2, nrounds = 2,objective = "binary:logistic" |
| Probability Recalibration | Isotonic regression | stats | isoreg |  |
| Interpretation machine learning | PDP&ICE | pdp | partial | ice = TRUE, grid.resolution = 40 |
| Model assessment | AUC | ROCR | performance | “measure=AUC”(so as it with other assessment indexes) |
